# Development of nifedipine isosteres: an integrated approach to the design, synthesis, and biological assessment of calcium channel blockers

**DOI:** 10.3389/fchem.2025.1581037

**Published:** 2025-05-12

**Authors:** Yasser M. Zohny, Samir M. Awad, Omar Alsaidan, Maha A. Rabie

**Affiliations:** ^1^ Pharmaceutical Chemistry department, College of Pharmacy, Shaqra University, Shaqra, Saudi Arabia; ^2^ Pharmaceutical Organic Chemistry Department, Faculty of Pharmacy, Helwan University, Cairo, Egypt; ^3^ Pharmacy Department, Al-Zahrawi University College, Karbala, Iraq; ^4^ Department of Pharmaceutics, College of Pharmacy, Jouf University, Sakaka, Al-Jouf, Saudi Arabia; ^5^ Pharmacology and Toxicology Department, College of Pharmacy, Shaqra University, Shaqra, Saudi Arabia; ^6^ Pharmacology and Toxicology Department, College of Pharmacy, Cairo University, Cairo, Egypt

**Keywords:** nifedipine isosteres, antihypertensive, calcium channel blocking, structure–activity relationship studies, docking

## Abstract

This study reports the synthesis of a series of calcium channel blockers via Biginelli’s reaction. The core dihydropyridine (DHP) scaffold, an isostere of nifedipine, was synthesized using three aldehydes incorporated with trifluoromethyl (–CF_3_) substitutions at the ortho, meta, and para positions. The resulting series **(4a–c** to **9a–c)** was evaluated for antihypertensive and calcium channel-blocking activities in male and female rats, administered intraperitoneally. Among the synthesized compounds, the ortho-substituted derivatives (**4a**, **7a**, **8a**, and **9a**) demonstrated the highest antihypertensive activity, exhibiting approximately 30% efficacy relative to nifedipine. These compounds also displayed IC_50_ values comparable to nifedipine and were further assessed for binding affinity with 6M7H and 4MS2 through molecular docking studies. The final DHP derivatives were amides, synthesized through reactions with aniline, 4-methylaniline, and 4-nitroaniline. Notably, compound **9a** exhibited the highest docking score against both tested receptor proteins, highlighting its potential for further investigation.

## 1 Introduction

Hypertension is a major risk factor for cardiovascular diseases, with key contributing factors including genetic predisposition, a sedentary lifestyle, obesity, and high salt intake. Calcium channel blockers (CCBs) play a crucial role in hypertension management by acting on L-type calcium channels in cardiac tissue and vascular smooth muscle. By inhibiting calcium influx, CCBs induce vasodilation, reduce peripheral vascular resistance, and lower blood pressure, making them effective as monotherapy for mild hypertension and as part of combination therapy for more severe cases (Jones et al., 2024; Aljehani et al., 2022). Compared to other antihypertensive drug classes, CCBs are generally well-tolerated and have fewer adverse effects (Volpe, 2018; Lip et al., 2022; Dharmarajan and Dharmarajan, 2015; Ojha et al., 2022).

Nifedipine, a well-established CCB, exerts its effects by blocking calcium influx through L-type calcium channels, leading to vasodilation and reduced myocardial oxygen demand (Shah et al., 2022). Beyond its cardiovascular benefits, studies suggest that nifedipine may also modulate immune responses, as experiments on colorectal cancer have demonstrated its ability to inhibit tumor growth by preventing NFAT2 nuclear translocation (Wu et al., 2020; Liang and Xiao, 2023). Additionally, long-acting formulations of nifedipine have been developed to improve patient compliance and minimize side effects, making it a preferred treatment for both angina and hypertension (Mavani et al., 2022). Although nifedipine has also been explored as a tocolytic agent, studies indicate no significant advantage in prolonging pregnancy compared to control groups (Olda et al., 2022).

Despite its widespread use, nifedipine has limitations, including dose-dependent side effects such as reflex tachycardia, flushing, and dizziness, necessitating the development of novel derivatives with improved pharmacological profiles.

This study aims to address these limitations by designing and synthesizing novel nifedipine isosteres, utilizing bioisosteric modifications to enhance calcium channel-blocking activity and improve therapeutic outcomes. Bioisosterism is a well-established strategy in drug design, allowing structural modifications that retain biological activity while optimizing efficacy, selectivity, and pharmacokinetic properties. In this work, we employ Biginelli’s reaction to synthesize a series of dihydropyridine (DHP)-based calcium channel blockers, incorporating trifluoromethyl (–CF_3_) substitutions at the ortho, meta, and para positions of benzaldehyde derivatives. These modifications are expected to influence molecular interactions with calcium channels, potentially enhancing binding affinity and activity.

A critical gap in current research lies in understanding how specific bioisosteric modifications impact calcium channel interactions at a molecular level. Existing DHP derivatives, including nifedipine and its analogs, have been extensively studied, but systematic structure–activity relationship (SAR) analyses focusing on trifluoromethyl-substituted derivatives remain limited. Our study seeks to bridge this gap by evaluating the antihypertensive efficacy of these novel compounds through *in vivo* experiments and molecular docking studies against known calcium channel receptor structures (6M7H and 4MS2).

By integrating synthetic chemistry, biological evaluation, and computational modeling, this research contributes to the rational design of next-generation calcium channel blockers with optimized therapeutic potential. The findings of this study may pave the way for the development of safer and more effective antihypertensive agents, addressing an unmet need in cardiovascular pharmacotherapy.

## 2 Materials and methods

### 2.1 Chemistry

The reactants and reagents used for the present study were obtained from Merck Pvt. Ltd., Darmstadt, Germany. The chemicals were used without any prior purifications. The Electrothermal IA 9100 equipment (Shimadzu, Japan) was used to measure the melting points of the target compounds. AMX-400 and Current AV400 Data spectrometers (400 MHz) (Bruker BioSpin GmbH, Germany) were used to measure the proton NMR spectra. The internal reference is trimethylsilane (TMS), and the changes in chemical shifts, δ, were expressed in parts per million. Using an MCA and polyethylene glycol (PEG) support, FAB high-resolution (HR) mass spectra were obtained using a VG Analytical 70-250S spectrometer in Palmer, United States, and a Finnigan Thermo Quest MAT 95XL spectrometer. The reactions were visualized by iodine vapors and UV rays and were tracked using thin-layer chromatography (TLC) with silica gel (60 F254)-coated aluminum plates (Merck). For column chromatography, 60–120-mesh silica gel was utilized.

#### 2.1.1 Synthesis of DHP ester derivatives

A mixture of thiourea (1) (7.6 g, 0.1 mol), ethyl acetoacetate (2) (13 mL, 0.1 mol), and the appropriate aromatic aldehyde (3) (0.1 mol) was added to a flask containing 50 mL of absolute ethanol and 1 mL of 37% HCl. The reaction mixture was refluxed for 8 h under continuous stirring. After completion, the mixture was cooled to room temperature and poured into an ice–water mixture, followed by neutralization with an ammonia solution. The resulting precipitate was collected by vacuum filtration, washed with cold ethanol, and dried under reduced pressure. The crude product was recrystallized from ethanol to afford derivatives **4a–c** in yields ranging from 70% to 75%.

**4a**: ethyl-6-methyl-2-thioxo-4-[2-(trifluoromethyl)phenyl]-1,2,3,4-tetrahydropyrimidine-5-carboxylate. Yield: 74%; m.p.: 261°C–263°C; IR ν (KBr cm^−1^): 3,432 (NH), 3,176 (CH, aromatic), 2,983 (CH, aliphatic), 1,753 (C=O ester), 1,685 (C=O), 1,270 (C=S), and 1,225 (C–O). ^1^H NMR (DMSO*d*_6_, 400 MHz) δ: 1.3 (t, 3H, CH_3_), 2.3 (s, 3H, CH_3_), 3.34 (q, 2H, CH_3_CH_2_–O), (s, 1H, CH), 7.28–9.6 (m, 4H, aromatic), and 9.67 [2H, 2NH (D_2_O exchangeable)]. ^13^C NMR: (DMSO*d*_6_, 400 MHz): δ 14.3 (1C, s), 18.6 (1C, s), 36.4 (1C, s), 54.9 (1C, s), 61.2 (1C, s), 99.5 (1C, s), 123.4 (1C, s), 127.2 (1C, s), 128.5–128.7 (2C), 128.6 (s), 128.6 (s), 130.3 (1C, s), 132.4 (1C, s), 145.7 (1C, s), 166.6 (1C, s), and 175.3 (1C, s). MS (EI) m/z: 344.35 (M^+^, 12.7%); Calcd./Anal., for C_15_H_15_F_3_N_2_O_2_S: C, 52.32; H, 4.39; N, 8.14. Found: C, 52.48; H, 4.28; N, 8.16.

**4b**: ethyl-6-methyl-2-thioxo-4-[3-(trifluoromethyl)phenyl]-1,2,3,4-tetrahydropyrimidine-5-carboxylate. Yield: 75%; m.p.: 277°C–279°C; IR ν (KBr cm^−1^): 3,445 (NH), 3,169 (CH, aromatic), 2,951 (CH, aliphatic), 1,759 (C=O ester), 1,680 (C=O), 1,276 (C=S), and 1,223(C–O). ^1^H NMR (DMSO*d*_6_, 400 MHz) δ: 1.19 (t, 3H, CH_3_), 2.3 (s, 3H, CH_3_), 3.99 (q, 2H, CH_3_CH_2_–O), 5.64 (s, 1H, CH), 7.36–8.26 (m, 4H, aromatic), 10.54, and 11.10 [s, 2H, 2NH (D_2_O exchangeable)]. ^13^C NMR: δ 14.3 (1C, s), 18.6 (1C, s), 39.5 (1C, s), 54.9 (1C, s), 61.2 (1C, s), 99.5 (1C, s), 123.8 (1C, s), 128.3–128.6 (2C), 128.4 (s), 128.5 (s), 129.4 (1C, s), 131.4 (1C, s), 139.5 (1C, s), 145.7 (1C, s), 166.6 (1C, s), and 175.3 (1C, s). MS (EI) m/z: 344.35 (M^+^, 15.8%); Calcd./Anal., for C_15_H_15_F_3_N_2_O_2_S: C, 52.32; H, 4.39; N, 8.14. Found: C, 52.35; H, 4.22; N, 8.13.

**4c**: ethyl-6-methyl-2-thioxo-4-[4-(trifluoromethyl)phenyl]-1,2,3,4-tetrahydropyrimidine-5-carboxylate. Yield: 70%; m.p.: 290°C–292°C; IR ν (KBr cm^−1^): 3,487 (NH), 3,170 (CH, aromatic), 2,958 (CH, aliphatic), 1,751 (C=O ester), 1,689 (C=O), 1,273 (C=S), and 1,227(C–O). ^1^H NMR (DMSO*d*_*6*_, 400 MHz) δ: 1.17 (t, 3H, CH_3_), 2.4 (s, 3H, CH_3_), 3.90 (q, 2H, CH_3_CH_2_–O), 5.70 (s, 1H, CH), 7.37–8.28 (m, 4H, aromatic), 10.52, and 11.13 [s, 2H, 2NH (D_2_O exchangeable)]. ^13^C NMR: δ 14.3 (1C, s), 18.6 (1C, s), 39.5 (1C, s), 54.9 (1C, s), 61.2 (1C, s), 99.5 (1C, s), 123.8 (1C, s), 129.4 (2C, s), 130.4 (2C, s), 134.7 (1C, s), 145.7 (1C, s), 166.6 (1C, s), and 175.3 (1C, s). MS (EI) m/z: 344.35 (M^+^, 10.4%); Calcd./Anal., for C_15_H_15_F_3_N_2_O_2_S: C, 52.32; H, 4.39; N, 8.14. Found: C, 52.39; H, 4.40; N, 8.19.

#### 2.1.2 Hydrolysis for the synthesis of carboxylic acid derivatives

A solution of **4a–c** (0.01 mol) in 50 mL of 10% alcoholic NaOH was refluxed for 2 h with continuous stirring. After cooling to room temperature, the reaction mixture was acidified with concentrated HCl, leading to the formation of a precipitate. The solid was collected by vacuum filtration, washed thoroughly with water, and dried under reduced pressure. The crude product was recrystallized from ethanol to afford derivatives **5a–c** in yields ranging from 60% to 67%.

**5a**: 6-methyl-2-thioxo-4-[2-(trifluoromethyl)phenyl]-1,2,3,4-tetrahydropyrimidine-5-carboxylic acid. Yield: 60%; m.p.: 245°C–247°C; ^1^H NMR (DMSO*d*_*6*_, 400 MHz) δ: 2.22 (s, 3H, CH_3_), 5.55 (s, 1H, CH), 7.25–8.18 (m, 4H, aromatic), 10.37, 11.19 [s, 2H, 2NH (D_2_O exchangeable)], and 11.8 [s, 1H, COOH (D_2_O exchangeable)]. ^13^C NMR: δ 18.6 (1C, s), 36.4 (1C, s), 54.9 (1C, s), 99.5 (1C, s), 123.4 (1C, s), 127.2 (1C, s), 128.5–128.7 (2C), 128.6 (s), 128.6 (s), 129.3 (1C, s), 132.4 (1C, s), 145.7 (1C, s), 167.1 (1C, s), and 175.3 (1C, s). MS (EI) *m*/*z*: 316.29 (M^+^, 11.6%); Calcd./Anal., for C_13_H_11_F_3_N_2_O_2_S: C, 49.36; H, 3.51; N, 8.86. Found: C, 49.26; H, 3.61; N, 8.72.

**5b**: 6-methyl-2-thioxo-4-[3-(trifluoromethyl)phenyl]-1,2,3,4-tetrahydropyrimidine-5-carboxylic acid*.* Yield: 64%; m.p.: 255°C–257°C; ^1^H NMR (DMSO*d*_6_, 400 MHz) δ: 2.23 (s, 3H, CH_3_), 5.51 (s, 1H, CH), 7.16–8.11 (m, 4H, aromatic), 10.26, 11.23 [s, 2H, 2NH (D_2_O exchangeable)], and 11.9 [s, 1H, COOH (D_2_O exchangeable)]. ^13^C NMR: δ 18.6 (1C, s), 39.5 (1C, s), 54.9 (1C, s), 99.5 (1C, s), 123.8 (1C, s), 128.3–128.6 (2C), 128.4 (s), 128.5 (s), 129.0 (1C, s), 131.4 (1C, s), 139.5 (1C, s), 145.7 (1C, s), 167.1 (1C, s), and 175.3 (1C, s). MS (EI) *m*/*z*: 316.29 (M^+^, 10.3%); Calcd./Anal., for C_13_H_11_F_3_N_2_O_2_S: C, 49.36; H, 3.51; N, 8.86. Found: C, 49.33; H, 3.42; N, 8.74.

**5c**: 6-methyl-2-thioxo-4-[4-(trifluoromethyl)phenyl]-1,2,3,4-tetrahydropyrimidine-5-carboxylic acid*.* Yield: 67%; m.p.: 264°C–266°C. ^1^H NMR (DMSO*d*_6_, 400 MHz) δ: 2.14 (s, 3H, CH_3_), 5.63 (s, 1H, CH), 7.18–8.15 (m, 4H, aromatic), 10.39, 11.17 [s, 2H, 2NH (D_2_O exchangeable)], 11.7 [s, 1H, COOH (D_2_O exchangeable)]. ^13^C NMR: δ 18.6 (1C, s), 39.5 (1C, s), 54.9 (1C, s), 99.5 (1C, s), 123.8 (1C, s), 129.4 (2C, s), 130.4 (2C, s), 130.9 (1C, s), 134.7 (1C, s), 145.7 (1C, s), 167.1 (1C, s), and 175.3 (1C, s). MS (EI) *m*/*z*: 316.29 (M^+^, 18.2%); Calcd./Anal., for C_13_H_11_F_3_N_2_O_2_S: C, 49.36; H, 3.51; N, 8.86. Found: C, 49.39; H, 3.60; N, 8.90.

#### 2.1.3 Synthesis of acid chloride derivatives

A mixture of **5a–c** (0.01 mol) and 15 mL of thionyl chloride was refluxed for 40 min with continuous stirring. Excess thionyl chloride was then removed by heating the reaction mixture on a water bath. The resulting acid chlorides **(6a–c)**, obtained in yields ranging from 59% to 68%, were rapidly dried under vacuum and used as crude intermediates for subsequent reactions.

**6a**: 6-methyl-2-thioxo-4-[2-(trifluoromethyl)phenyl]-1,2,3,4-tetrahydropyrimidine-5-carbonyl chloride. Yield: 60%; m.p.: 269°C–271°C. ^1^H NMR (DMSO*d*_6_, 400 MHz) δ: 2.11 (s, 3H, CH_3_), 5.57 (s, 1H, CH), 7.11–8.13 (m, 4H, aromatic), 10.23, and 11.18 [s, 2H, 2NH (D_2_O exchangeable)]. ^13^C NMR: δ 18.6 (1C, s), 36.4 (1C, s), 54.9 (1C, s), 99.5 (1C, s), 123.4 (1C, s), 127.2 (1C, s), 128.5–128.7 (2C), 128.6 (s), 128.6 (s), 132.4 (1C, s), 145.7 (1C, s), 175.3 (1C, s), and 176.8 (1C, s). MS (EI) *m*/*z*: 334.74 (M^+^, 11.5%); Calcd./Anal., for C_13_H_10_ClF_3_N_2_OS: C, 46.64; H, 3.01; N, 8.37. Found: C, 46.71; H, 3.10; N, 8.40.

**6b**: 6-methyl-2-thioxo-4-[3-(trifluoromethyl)phenyl]-1,2,3,4-tetrahydropyrimidine-5-carbonyl chloride. Yield: 68%; m.p.: 281°C–283°C. ^1^H NMR (DMSO*d*_6_, 400 MHz) δ: 2.14 (s, 3H, CH_3_), 5.50 (s, 1H, CH), 7.15–8.17 (m, 4H, aromatic), 10.03, and 11.27 [s, 2H, 2NH (D_2_O exchangeable)]. ^13^C NMR: δ 18.6 (1C, s), 39.5 (1C, s), 54.9 (1C, s), 99.5 (1C, s), 123.8 (1C, s), 128.3–128.6 (2C), 128.4 (s), 128.5 (s), 129.0 (1C, s), 139.5 (1C, s), 145.7 (1C, s), 175.3 (1C, s), and 176.8 (1C, s). MS (EI) *m*/*z*: 334.74 (M^+^, 7.8%); Calcd./Anal., for C_13_H_10_ClF_3_N_2_OS: C, 46.64; H, 3.01; N, 8.37. Found: C, 46.75; H, 3.11; N, 8.32.

**6c**: 6-methyl-2-thioxo-4-[4-(trifluoromethyl)phenyl]-1,2,3,4-tetrahydropyrimidine-5-carbonyl chloride. Yield: 59%; m.p.: 275°C–277°C. ^1^H NMR (DMSO*d*_6_, 400 MHz) δ: 2.25 (s, 3H, CH_3_), 5.58 (s, 1H, CH), 7.31–8.29 (m, 4H, aromatic), 10.16, and 11.28 [s, 2H, 2NH (D_2_O exchangeable)].^13^C NMR: δ 18.6 (1C, s), 39.5 (1C, s), 54.9 (1C, s), 99.5 (1C, s), 123.8 (1C, s), 129.4 (2C, s), 130.4 (2C, s), 134.7 (1C, s), 145.7 (1C, s), and 176.8 (1C, s). MS (EI) *m*/*z*: 334.74 (M^+^, 9.7%); Calcd./Anal., for C_13_H_10_ClF_3_N_2_OS: C, 46.64; H, 3.01; N, 8.37. Found: C, 46.69; H, 3.07; N, 8.39.

#### 2.1.4 Synthesis of amide derivatives

A mixture of **6a–c** (0.01 mol) and the appropriate aromatic amine (0.01 mol) in 25 mL of ethanol was refluxed for 5 h under continuous stirring. After cooling to room temperature, the resulting precipitate was collected by filtration, dried under vacuum, and recrystallized from ethanol to yield derivatives **7a–c**, **8a–c**, and **9a–c** in yields ranging from 65% to 77%.

**7a**: N-phenyl-6-methyl-2-thioxo-4-[2-(trifluoromethyl)phenyl]-1,2,3,4-tetrahydropyrimidine-5-carboxamide. Yield: 70%; m.p.: 290°C–292°C. ^1^H NMR (DMSO*d*_6_, 400 MHz) δ: 2.34 (s, 3H, CH_3_), 5.57 (s, 1H, CH), 7.35–8.43 (m, 9H, aromatic), 10.17, 10.5, and 11.33 [s, 3H, 3NH (D_2_O exchangeable)]. ^13^C NMR: δ 18.6 (1C, s), 36.4 (1C, s), 54.9 (1C, s), 99.5 (1C, s), 119.1 (2C, s), 123.4 (1C, s), 127.2 (1C, s), 128.5–128.7 (4C), 128.6 (s), 128.6 (s), 128.6 (s), 129.3 (1C, s), 130.3 (1C, s), 132.4 (1C, s), 137.5 (1C, s), 145.7 (1C, s), 163.8 (1C, s), and 175.3 (1C, s). MS (EI) *m*/*z*: 391.41 (M^+^, 13.7%); Calcd./Anal., for C_19_H_16_F_3_N_3_OS: C, 58.30; H, 4.12; N, 10.74. Found: C, 58.29; H, 4.07; N, 10.81.

**7b**: N-(4-methylphenyl)-6-methyl-2-thioxo-4-[2-(trifluoromethyl)phenyl]-1,2,3,4-tetrahydropyrimidine-5-carboxamide. Yield: 75%; m.p.: 287°C–289°C. ^1^H NMR (DMSO*d*_6_, 400 MHz) δ: 2.3, 2.5 (s, 6H, 2CH_3_), 5.53 (s, 1H, CH), 7.45–8.48 (m, 8H, aromatic), 10.18, 10.54, and 11.31 [s, 3H, 3NH (D_2_O exchangeable)]. ^13^C NMR: δ 18.6 (1C, s), 36.4 (1C, s), 54.9 (1C, s), 55.9 (1C, s), 99.5 (1C, s), 114.6 (2C, s), 119.0 (2C, s), 123.4 (1C, s), 127.2 (1C, s), 128.5–128.7 (2C), 128.6 (s), 128.6 (s), 130.3 (1C, s), 132.4 (1C, s), 137.5 (1C, s), 145.7 (1C, s), 159.7 (1C, s), 163.8 (1C, s), and 175.3 (1C, s). MS (EI) *m*/*z*: 405.43(M^+^, 19.2%); Calcd./Anal., for C_20_H_18_F_3_N_3_OS: C, 59.29; H, 4.47.36; N, 10.36. Found: C, 59.31; H, 4.37; N, 10.32.

**7c**: N-(4-nitrophenyl)-6-methyl-2-thioxo-4-[2-(trifluoromethyl)phenyl]-1,2,3,4-tetrahydropyrimidine-5-carboxamide. Yield: 77%; m.p.: 297°C–299°C. ^1^H NMR (DMSO*d*_6_, 400 MHz) δ: 2.32 (s, 3H, CH_3_), 5.58 (s, 1H, CH), 7.43–8.45 (m, 8H, aromatic), 10.13, 10.54, and 11.32 [s, 3H, 3NH (D_2_O exchangeable)]. ^13^C NMR: δ 18.6 (1C, s), 36.4 (1C, s), 54.9 (1C, s), 99.5 (1C, s), 114.2 (2C, s), 119.0 (2C, s), 123.4 (1C, s), 127.2 (1C, s), 128.5–128.7 (2C), 128.6 (s), 128.6 (s), 130.3 (1C, s), 132.4 (1C, s), 137.5 (1C, s), 145.7 (1C, s), 148.3 (1C, s), 163.8 (1C, s), and 175.3 (1C, s). MS (EI) *m*/*z*: 391.41 (M^+^, 10.7%); Calcd./Anal., for C_19_H_15_F_3_N_4_O_3_S: C, 52.29; H, 3.46; N, 12.84. Found: C, 52.30; H, 3.43; N, 12.81.

**8a**: N-phenyl-6-methyl-2-thioxo-4-[3-(trifluoromethyl)phenyl]-1,2,3,4-tetrahydropyrimidine-5-carboxamide. Yield: 68%; m.p.: 298°C–300°C. ^1^H NMR (DMSO*d*_6_, 400 MHz) δ: 2.34 (s, 3H, CH_3_), 5.62 (s, 1H, CH), 7.37–8.05 (m, 9H, aromatic), 10.18, 10.5, and 11.35 [s, 3H, 3NH (D_2_O exchangeable)]. ^13^C NMR: δ 18.6 (1C, s), 39.5 (1C, s), 54.9 (1C, s), 99.5 (1C, s), 119.1 (2C, s), 123.8 (1C, s), 128.3–128.7 (4C), 128.4 (s), 128.5 (s), 128.6 (s), 129.0 (1C, s), 129.4 (1C, s), 131.4 (1C, s), 137.5 (1C, s), 139.5 (1C, s), 145.7 (1C, s), 163.8 (1C, s), and 175.3 (1C, s). MS (EI) *m*/*z*: 391.41 (M^+^, 15.3%); Calcd./Anal., for C_19_H_16_F_3_N_3_OS: C, 58.30; H, 4.12; N, 10.74. Found: C, 58.35; H, 4.03; N, 10.68.

**8b**: N-(4-methylphenyl)-6-methyl-2-thioxo-4-[3-(trifluoromethyl)phenyl]-1,2,3,4-tetrahydropyrimidine-5-carboxamide. Yield: 70%; m.p.: 305°C–307°C; ^1^H NMR (DMSO*d*_6_, 400 MHz) δ: 2.4, 2.6 (s, 6H, 2CH_3_), 5.52 (s, 1H, CH), 7.50–8.13 (m, 8H, aromatic), 10.18, 10.61, 11.31 [s, 3H, 3NH (D_2_O exchangeable)]. ^13^C NMR: δ 18.6 (1C, s), 39.5 (1C, s), 54.9 (1C, s), 55.9 (1C, s), 99.5 (1C, s), 114.6 (2C, s), 119.0 (2C, s), 123.8 (1C, s), 128.3–128.6 (2C), 128.4 (s), 128.5 (s), 129.4 (1C, s), 131.4 (1C, s), 137.5 (1C, s), 139.5 (1C, s), 145.7 (1C, s), 159.7 (1C, s), 163.8 (1C, s), and 175.3 (1C, s). MS (EI) *m*/*z*: 405.43(M+, 17.6%); Calcd./Anal., for C_20_H_18_F_3_N_3_OS: C, 59.29; H, 4.47.36; N, 10.36. Found: C, 59.37; H, 4.42; N, 10.41.

**8c**: N-(4-nitrophenyl)-6-methyl-2-thioxo-4-[3-(trifluoromethyl)phenyl]-1,2,3,4-tetrahydropyrimidine-5-carboxamide. Yield: 70%; m.p.: 286°C–288°C. ^1^H NMR (DMSO*d*_6_, 400 MHz) δ: 2.35 (s, 3H, CH_3_), 5.57 (s, 1H, CH), 7.36–8.76 (m, 8H, aromatic), 10.18, 10.53, and 11.22 [s, 3H, 3NH (D_2_O exchangeable)]. ^13^C NMR: δ 18.6 (1C, s), 39.5 (1C, s), 54.9 (1C, s), 99.5 (1C, s), 114.2 (2C, s), 119.0 (2C, s), 123.8 (1C, s), 128.3–128.6 (2C), 128.4 (s), 128.5 (s), 129.0 (1C, s), 129.4 (1C, s), 131.4 (1C, s), 137.5 (1C, s), 145.7 (1C, s), 148.3 (1C, s), 163.8 (1C, s), and 175.3 (1C, s). MS (EI) *m*/*z*: 391.41 (M^+^, 10.7%); Calcd./Anal., for C_19_H_15_F_3_N_4_O_3_S: C, 52.29; H, 3.46; N, 12.84. Found: C, 52.19; H, 3.39; N, 12.87.

**9a**: N-phenyl-6-methyl-2-thioxo-4-[4-(trifluoromethyl)phenyl]-1,2,3,4-tetrahydropyrimidine-5-carboxamide. Yield: 65%; m.p.: 273°C–275°C. ^1^H NMR (DMSO*d*_6_, 400 MHz) δ: 2.38 (s, 3H, CH_3_), 5.64 (s, 1H, CH), 7.41–8.06 (m, 9H, aromatic), 10.23, 10.4, and 11.33 [s, 3H, 3NH (D_2_O exchangeable)]. ^13^C NMR: δ 18.6 (1C, s), 39.5 (1C, s), 54.9 (1C, s), 99.5 (1C, s), 119.1 (2C, s), 123.8 (1C, s), 128.6 (2C, s), 129.4 (2C, s), 130.4 (2C, s), 130.9 (1C, s), 134.7 (1C, s), 137.5 (1C, s), 145.7 (1C, s), 163.8 (1C, s), and 175.3 (1C, s). MS (EI) *m*/*z*: 391.41 (M^+^, 11.8%); Calcd./Anal., for C_19_H_16_F_3_N_3_OS: C, 58.30; H, 4.12; N, 10.74. Found: C, 58.43; H, 4.17; N, 10.73.

**9b**: N-(4-methylphenyl)-6-methyl-2-thioxo-4-[4-(trifluoromethyl)phenyl]-1,2,3,4-tetrahydropyrimidine-5-carboxamide. Yield: 70%; m.p.: 309°C–311°C. ^1^H NMR (DMSO*d*_6_, 400 MHz) δ: 2.5, 2.8 (s, 6H, 2CH_3_), 5.51 (s, 1H, CH), 7.61–8.14 (m, 8H, aromatic), 10.23, 10.66, and 11.72 [s, 3H, 3NH (D_2_O exchangeable)]. ^13^C NMR: δ 18.6 (1C, s), 39.5 (1C, s), 54.9 (1C, s), 55.9 (1C, s), 99.5 (1C, s), 114.6 (2C, s), 119.0 (2C, s), 123.8 (1C, s), 129.4 (2C, s), 130.4 (2C, s), 134.7 (1C, s), 137.5 (1C, s), 145.7 (1C, s), 159.7 (1C, s), 163.8 (1C, s), and 175.3 (1C, s). MS (EI) *m*/*z*: 405.43(M^+^, 10.7%); Calcd./Anal., for C_20_H_18_F_3_N_3_OS: C, 59.29; H, 4.47.36; N, 10.36. Found: C, 59.24; H, 4.51; N, 10.38.

**9c**: N-(4-nitrophenyl)-6-methyl-2-thioxo-4-[4-(trifluoromethyl)phenyl]-1,2,3,4-tetrahydropyrimidine-5-carboxamide. Yield: 75%; m.p.: 290°C–292°C. ^1^H NMR (DMSO*d*_6_, 400 MHz) δ: 2.75 (s, 3H, CH_3_), 5.61 (s, 1H, CH), 7.39–8.52 (m, 8H, aromatic), 10.19, 10.48, and 11.36 [s, 3H, 3NH (D_2_O exchangeable)]. ^13^C NMR: δ 18.6 (1C, s), 39.5 (1C, s), 54.9 (1C, s), 99.5 (1C, s), 114.2 (2C, s), 119.0 (2C, s), 123.8 (1C, s), 129.4 (2C, s), 130.4 (2C, s), 134.7 (1C, s), 137.5 (1C, s), 145.7 (1C, s), 148.3 (1C, s), 163.8 (1C, s), and 175.3 (1C, s). MS (EI) *m*/*z*: 391.41 (M^+^, 13.9%); Calcd./Anal., for C_19_H_15_F_3_N_4_O_3_S: C, 52.29; H, 3.46; N, 12.84. Found: C, 52.29; H, 3.51; N, 12.78.

### 2.2 Biological evaluation

#### 2.2.1 *In vivo* antihypertensive studies

Heparin was administered intraperitoneally (i.p.) at a dose of 2,000 IU/kg to rats of both sexes to prevent blood clotting. Anesthesia was induced by injecting pentothal sodium (80 mg/kg, i.p.) to ensure minimal distress during the procedure. A mercury manometer was used to calibrate the blood pressure transducer before each experiment. The carotid artery of each rat was carefully cannulated using a polyethylene catheter (PE-50) prefilled with heparinized saline (50 IU/mL) and connected to a blood pressure transducer to continuously monitor arterial blood pressure. The transducer was linked to a data acquisition system for real-time recording. To evaluate the effect of venous flow on blood pressure and suppress the adrenaline response, a second catheter (0.3 mL of heparinized saline) was inserted into the jugular vein on the contralateral side. Baseline blood pressure readings were recorded before administering the test compounds which were injected intraperitoneally at 2 mg/mL (0.3 mL in volume) solution.

#### 2.2.2 CCB activity

The calcium channel-blocking activity of the test compounds was evaluated using isolated rat ileum preparations in an organ bath setup. The organ bath (50 mL capacity) was filled with a slightly modified Tyrode solution, composed of the following constituents: NaCl = 8.0 gm/L; KCl = 0.2 g/L; CaCl_2_ = 0.18 g/L; NaH_2_PO_4_ = 0.1 g/L; MgCl_2_ = 0.1 g/L; glucose = 1.0 g/L; and NaHCO_3_ = 1.0 g/L. The solution was continuously aerated with a 95% O_2_ and 5% CO_2_ mixture and maintained at 37°C to simulate physiological conditions. To induce ileum contraction, the bath was supplemented with potassium chloride and calcium chloride.

##### 2.2.2.1 Tissue preparation

Rats were fasted overnight prior to the experiment to prevent interference from food metabolites. Euthanasia was performed via a sharp blow to the head, followed by cervical dislocation and severing of the neck blood vessels. Immediately after opening the abdominal cavity, an approximately 2–3-cm segment of the ileum was carefully isolated and placed in a Petri dish containing pre-warmed (37°C) Tyrode solution. The mesentery was carefully removed, and the lumen of the ileum was flushed with Tyrode solution using a pipette to remove any residual contents.

The cleaned ileum segment was then mounted in the organ bath and connected to an isotonic frontal writing lever. The tissue was allowed to equilibrate for 30 min with regular washing using fresh Tyrode solution every 10 min to maintain tissue responsiveness.

##### 2.2.2.2 Experimental protocol

To establish a baseline contraction response, acetylcholine (Ach) was added to the organ bath at incremental concentrations until the maximum contractile effect was observed. The bath was then emptied, and fresh Tyrode solution containing the test compound (2 mg/mL, 0.3 mL) was introduced. After incubation with the test compound, the same amount of acetylcholine was re-administered, and the contractile response was measured.

The degree of muscle relaxation induced by the test compounds was recorded and compared to the pre-contracted state. The percentage inhibition of contraction was calculated for each concentration of the test compound. The IC_50_ value (the concentration required to achieve 50% relaxation) was determined using linear regression analysis. The calculation followed the equation:

If y = 50%, then x = 0.5 mL dose,

where x represents the dose required to produce 50% inhibition of contraction.

### 2.3 Molecular docking studies

Based on the pharmacological results, we selected compounds **4a**, **7a**, **8a**, and **9a**, the inhibitors in this study, as the docking model (PDB IDs: 6M7H, and 4MS2) (Johnson et al., 2019; Tang et al., 2014). Computer-guided docking experiments were conducted using Molecular Operating Environment (MOE 2015.10) software, Chemical Computing Group, Montreal, Canada. Molecular docking studies were conducted to get a deeper insight into the molecular bases of the inhibitory potency for lead optimization and to pick up the interaction between compounds and the ryanodine receptor.

## 3 Results and discussion

### 3.1 Chemistry

**SCHEME 1:**
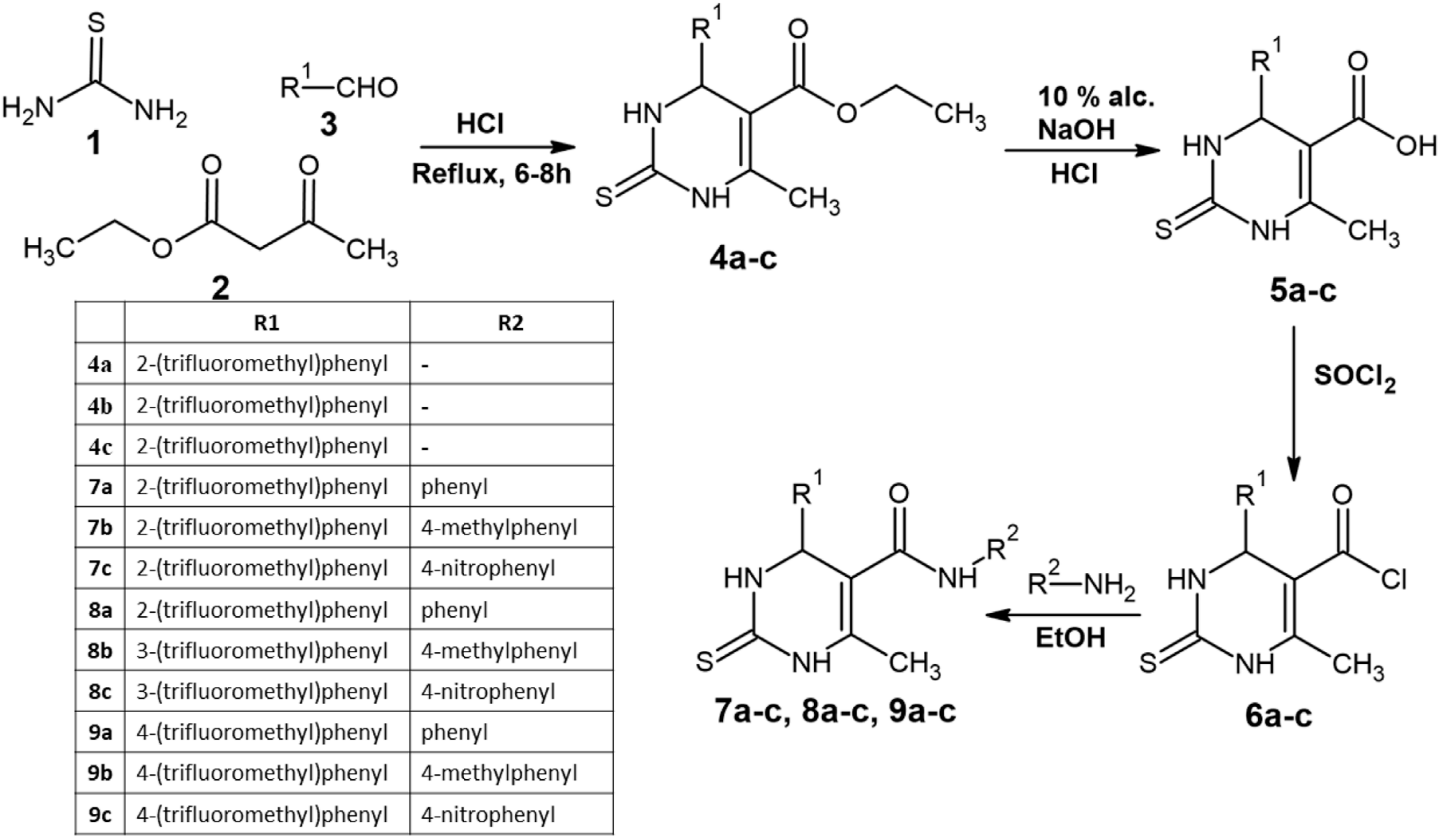
Reaction scheme for the synthesized compounds.

The synthetic pathway for DHPs in this study is illustrated in Scheme 1. It has been reported that structural modifications to the DHP ring, particularly the introduction of bulky substituents at specific positions, can significantly enhance its activity, with some derivatives reported to exhibit up to three times the potency of nifedipine (Shaldam et al., 2016). In this work, the synthesized compounds share a bioisosteric core with nifedipine. Specifically, within the dihydropyrimidine ring, the two nitrogen (N) atoms act as bioisosteres for carbon–hydrogen (CH) groups, whereas the methyl (-CH_3_) group serves as a bioisostere for the ketone (C=O) found in the DHP ring of nifedipine. Additionally, the ester (–COO–) linkage present in nifedipine has been replaced with an amide (–CONH–) linkage in the test compounds, a modification that may alter their pharmacological properties.

The synthesis of the target compounds began with the condensation (1 and 2) with trifluoromethyl-substituted aldehydes at the ortho, meta, and para positions (3) in an acidic medium, leading to the cyclization of the thiopyrimidine ring (**4a–c**). The yields for **4a, 4b, and 4c** were 74%, 75%, and 70%, respectively. The formation of the cyclized thiopyrimidine ring was confirmed by ^1^H NMR peaks at 7–8 ppm and aromatic C–H IR peaks at 3,176, 3,169, and 3,170 cm^−1^.

The **4a–c** series then underwent hydrolysis, converting carboxylates into carboxylic acid groups, yielding compounds **5a–c**. The presence of a ^1^H NMR peak at 11.8 ppm confirmed successful hydrolysis. The carboxylic acid groups were subsequently transformed into acid chlorides via thionyl chloride (SOCl_2_) treatment, resulting in compounds **6a–c**. This modification increased the reactivity of the compounds, making them suitable for further derivatization with amines.

The final series of compounds (**7a–c**, **8a–c**, and **9a–c**) were synthesized by reacting **6a–c** with three different amines—aniline, 4-methylaniline, and 4-nitroaniline—via nucleophilic substitution in ethanol, forming an amide linkage at the fifth position of the thiopyrimidine ring. The substituent R_2_ was phenyl, 4-methylphenyl, and 4-nitrophenyl, corresponding to the **a, b**, and **c** series of compounds, respectively.

### 3.2 Biological evaluation

#### 3.2.1 Antihypertensive ability

The choice of aldehydes significantly influences the Biginelli’s reaction pathway and the nature of the final products. Different aldehydes can lead to distinct chemical routes, affecting product composition. For instance, aldol condensation between 2,4-pentanedione and various aldehydes has been reported to yield diverse compounds, such as dimethylbicyclo [3.3.1]nonadienediones (Sekiya et al., 1973). Moreover, the reactivity of the final compounds is influenced by the chemical versatility of the aldehydes used. In this study, aromatic aldehydes were selected, with a –CF_3_ group substituted at the ortho, meta, and para positions of benzaldehyde. The impact of these substitutions on antihypertensive and calcium channel-blocking (CCB) activity was systematically evaluated.

The synthesized compounds **(4a–c**, **5a–c**, **6a–c**, **7a–c**, **8a–c**, and **9a–c**) were administered to rats at a fixed dose, and blood pressure measurements were taken before and after administration. The percentage inhibition in blood pressure was then calculated and summarized in Table 1. As a reference, nifedipine, a well-established antihypertensive agent, exhibited 27.35%–30.43% inhibition. Among the **4a–c** series, inhibition ranged from 18.46% to 30.73%, with **4a** (ortho-substituted trifluoromethyl) demonstrating a slightly better antihypertensive effect than nifedipine. Further comparative analysis of blood pressure inhibition suggested that compounds **7a**, **8a**, and **9a** displayed superior antihypertensive activity, making them promising candidates for further investigation.

**TABLE 1:** Antihypertensive activity of nifedipine and the synthesized compounds following an intraperitoneal injection of 2 mg/mL (0.3 mL in volume).

Compound code	Control (mm Hg)	Test (mm Hg)	% Inhibition in blood pressure
Nifedipine	29.33 28.28	20.17 20.78	30.43 27.35
**4a**	28.41 27.57	21.08 20.63	30.73 28.52
**4b**	28.48 28.38	23.76 24.27	18.65 18.79
**4c**	29.73 28.28	22.18 22.28	18.46 18.63
**5a**	29.88 29.15	25.17 25.74	15.72 15.12
**5b**	30.01 29.29	24.21 24.14	16.23 16.93
**5c**	28.19 28.32	20.35 20.42	30.32 29.08
**6a**	30.23 30.02	26.54 26.23	11.75 11.79
**6b**	28.90 28.48	26.85 26.92	11.09 11.17
**6c**	30.12 30.01	27.45 27.09	11.00 11.02
**7a**	29.32 30.15	20.19 21.42	31.42 27.58
**7b**	29.19 30.01	21.98 21.86	23.23 23.90
**7c**	29.22 30.02	22.02 22.19	24.12 24.23
**8a**	30.48 29.48	22.62 21.03	26.70 30.23
**8b**	30.51 30.00	25.61 25.03	22.21 22.81
**8c**	29.04 29.61	25.18 22.90	22.32 22.73
**9a**	29.15 30.17	22.25 22.73	25.37 26.07
**9b**	30.10 30.71	24.28 24.45	23.32 23.97
**9c**	29.71 30.47	24.23 24.84	23.34 23.75

#### 3.2.2 CCB activity

The CCB activity of the synthesized compounds is provided in Table 2. Nifedipine as the standard showed a dose-dependent activity, where the % inhibition increased with the dose, and at a dose of 0.6 ml, 61.56% inhibition was observed with an IC_50_ of 21. Compounds **4b** and **4c** exhibited lesser potency than **4a**. Consistent inhibition is obtained for series **5** compounds. Compound **5a** showed 35.23% inhibition at 0.5 ml and has an IC_50_ of 24.37, which is in the same range as nifedipine. The compounds **6a–c** showed weaker activity; at 0.1 mL dosage, the inhibition was lower (∼8%), but with increased dose, the inhibition reached approximately 30%. Compared to the previous set of compounds, compound **7a** showed nearly 50% inhibition at 0.5 mL dosage, making it quite effective. Compound **7c** has also shown a good inhibition profile, with 44.98% at 0.5 mL and the IC_50_ of 22.85, which is closer to those of nifedipine. Similar to series **7** compounds, series **8** compounds also showed moderate to good efficiency. Compounds **9a–c** at 0.5 mL also showed consistent and strong inhibition. The results conclude that the compounds with ortho substitution have shown better inhibition than meta and para substitutions, which is owed to several factors.

**TABLE 2:** Screening of CCB activity screened for the synthesized compounds compared to nifedipine at a concentration of 2 mg/mL.

Compound code	Dose (ml)	Control (cm)	Test (cm)	% Inhibition	IC_50_
Nifedipine	0.1	3.4	3.0	11.74	21
	0.2	3.4	2.7	20.47	
	0.3	3.4	2.3	32.26	
	0.4	3.3	2.1	35.76	
	0.5	3.3	1.7	48.38	
	0.6	3.3	1.2	61.56	
**4a**	0.1	3.4	2.9	17.24	19.07
	0.3	3.4	2.3	20.56	
	0.5	3.3	1.8	30.80	
**4b**	0.1	3.4	2.8	16.78	24.69
	0.3	3.4	2.3	19.36	
	0.5	3.3	1.6	29.83	
**4c**	0.1	3.3	2.7	17.25	23.62
	0.3	3.4	2.3	21.19	
	0.5	3.4	1.6	32.97	
**5a**	0.1	3.4	2.6	14.88	24.37
	0.3	3.3	2.1	19.71	
	0.5	3.4	1.6	35.23	
**5b**	0.1	3.3	2.7	14.87	25.25
	0.3	3.4	2.3	19.98	
	0.5	3.4	1.8	34.89	
**5c**	0.1	3.4	2.7	13.67	20.83
	0.3	3.4	2.3	18.96	
	0.5	3.3	1.5	32.96	
**6a**	0.1	3.3	2.6	8.56	26.84
	0.3	3.4	2.3	17.87	
	0.5	3.4	1.8	30.84	
**6b**	0.1	3.4	2.7	7.98	30.78
	0.3	3.3	2.1	16.98	
	0.5	3.4	1.8	30.09	
**6c**	0.1	3.3	2.7	8.98	29.73
	0.3	3.4	2.1	17.43	
	0.5	3.4	1.5	30.53	
**7a**	0.1 0.3 0.5	3.4 3.4 3.4	2.8 2.2 1.7	17.64 35.29 50.00	19.65
**7b**	0.1	3.4	2.6	16.09	22.81
	0.3	3.4	2.2	30.93	
	0.5	3.3	1.8	44.89	
**7c**	0.1	3.4	2.7	15.96	22.85
	0.3	3.4	2.2	28.97	
	0.5	3.3	1.5	44.98	
**8a**	0.1 0.3 0.5	3.4 3.4 3.4	2.7 2.1 1.5	16.24 30.23 49.10	20.23
**8b**	0.1	3.3	2.7	16.76	22.91
	0.3	3.4	2.1	29.56	
	0.5	3.4	1.5	44.87	
**8c**	0.1	3.3	2.7	16.76	22.63
	0.3	3.4	2.1	28.98	
	0.5	3.4	1.5	45.83	
**9a**	0.1 0.3 0.5	3.4 3.4 3.3	2.6 2.01 1.4	16.12 32.45 48.75	21.45
**9b**	0.1 0.3 0.5	3.4 3.4 3.3	2.8 2.1 1.3	15.81 30.53 42.82	22.43
**9c**	0.1 0.3 0.5	3.3 3.5 3.5	2.5 2.3 1.8	15.76 33.20 43.91	22.48

The considered aldehydes possessed a –CF_3_ group positioned at the ortho, meta, and para positions to the –CHO, which is further attached to the DHP ring in the preceding steps. Since the CCB activity is majorly because of the DHP ring, it would result in the varied responses for the substituted aldehydes. The ortho substitution, where the group is placed directly adjacent (close proximity) to the functional groups responsible for CCB, would influence the overall conformation of the molecule, allowing it to have better interaction with the calcium channel. In contrast, meta and para substitutions are further away from the active sites of the molecule, resulting in less favorable interactions with calcium channels. Among the final compounds **7**(a–c) to **9**(a–c), the ‘a’ series forming an amide linkage with an aid of aniline presented greater CCB activity. Compounds possessing –CH_3_ and –NO_2_ groups on a phenyl group showed a lesser docking score attributing to their corresponding electron-donating and electron-withdrawing nature, affecting the electron density on the nitrogen of amide linkage.

### 3.3 Molecular docking studies

The compounds **4a**, **7a**, **8a**, and **9a** were docked at the binding sites of 6M7H and 4MS2 receptors. The binding affinities were evaluated based on the binding free energy S-score and hydrogen bonds with their distance between the designed compounds and the amino acids in the receptor (Table 3). A total of 15 residues were found at the binding of the 4MS2 receptor, whereas 252 residues were seen in the 6M7H receptor.

**TABLE 3:** Summary of Molecular Operating Environment (MOE) docking results for synthesized compounds with target proteins.

Compound ID		Docking score	Ligand	Receptor	Interaction	Bond length	Energy	Interacting residues
6M7H								
**4a**		−4.9	N2	SE	H-donor	4.37	−0.6	MSE71
**7a**		−5.7	S20	CB	H-acceptor	3.35	−0.6	Glu84
**8a**		−4.8	C31	OE1	H-donor	2.91	−0.8	Glu84
**9a**		−5.7	N6	OE1	H-donor	2.36	−0.7	Glu84
Standard		−5.8	C 25	OE1	H-donor	2.83	−1.5	Glu84
KN9		−7.3	C18 20 O2 60 O3 62 N1 56	SD CE CE OD2	H-donor H-acceptor H-acceptor Ionic	4.16 3.42 3.31 3.71	−0.9 −0.6 −0.9 −1.2	Met71 Lys75 Lys75 Asp80
4MS2								
**4a**		−3.6	S20 S20	CA OG1	H-acceptor H-acceptor	3.35 3.76	−1.4 −1.3	Thr1206 Thr1206
**7a**		−6.2	S20 S20	CA OG1	H-acceptor H-acceptor	2.98 3.94	−3.95 −3.77	Thr1206 Thr1206
**8a**		−3.6	F42	NE2	H-acceptor	2.72	−0.7	Gln1150
**9a**		−6.3	6-ring	CG2	Pi–H	3.97	−0.6	Thr1206
Standard		−5.7	O8	CA	H-acceptor	3.62	−0.9	Thr1175
PX4		−7.7	O1 1 O1 1 O2 2 O2 2	CA N NH1 NH1	H-acceptor H-acceptor H-acceptor Ionic	3.5 3.03 3.19 3.19	−0.6 −5.4 −0.8 −3.3	Pro1090 Thr1091 Arg1102 Arg1102

**FIGURE 1:**
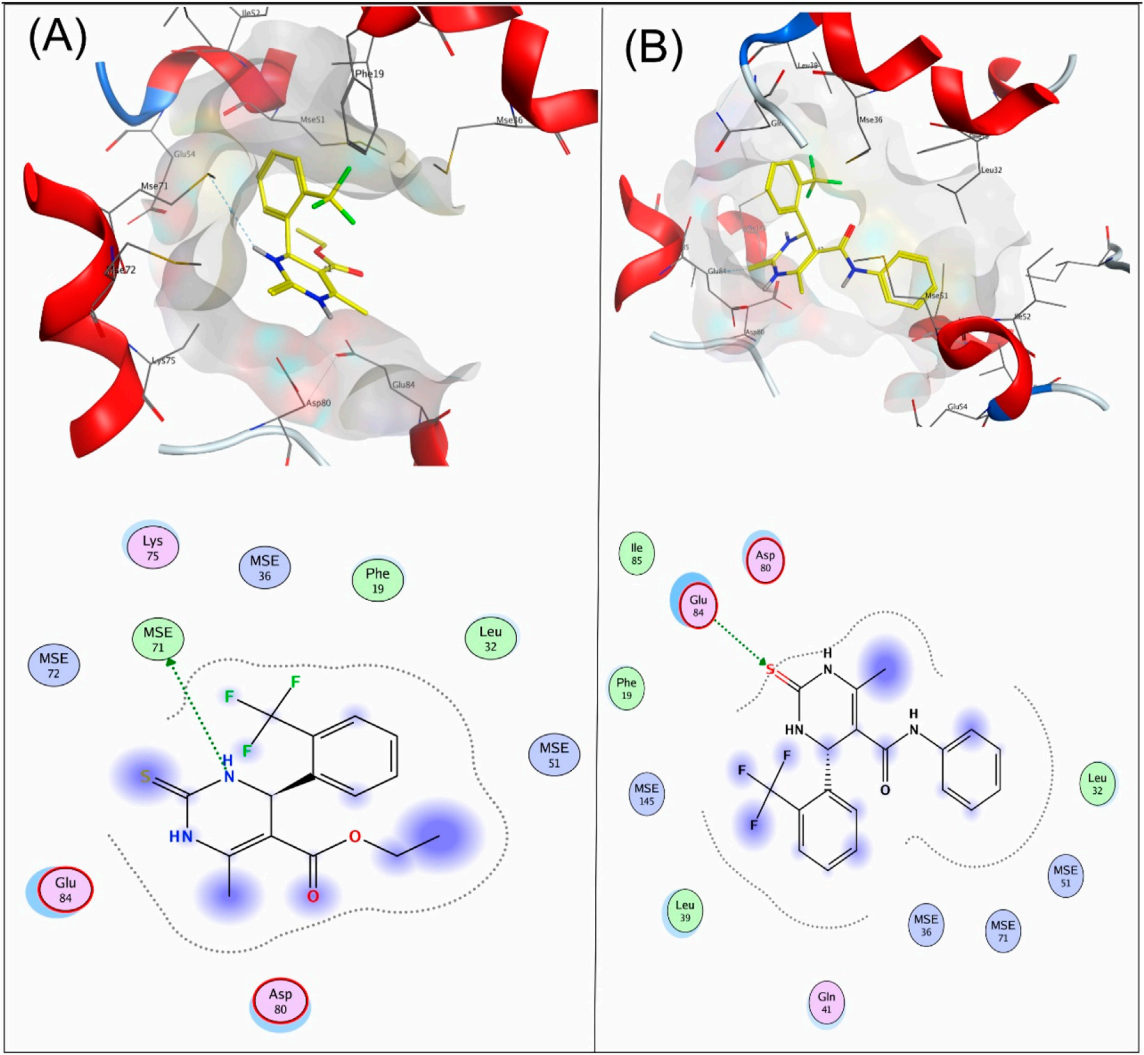
**(A)** Interaction of compound **4a** and **(B)** compound **7a** with the target protein 6M7H.

Compound **4a** showed a docking score of −4.9, having one H-donor interaction with the MSE71 residue of the target receptor 6M7H catalytic active binding site, as shown in Figure 1A. Compound **7a** showed a −5.7 docking score with H-acceptor interaction involving Glu84 and the sulfur group of the ligand, as shown in Figure 1B.

### 3.4 Structure–activity relationships

The analysis of the structure–activity relationships shows that the addition of different aromatic rings to the 6-methyl-2-thioxo-1,2,3,4-tetrahydropyrimidine-5-yl methanone moiety resulted in variation in the calcium channel-blocking activity of these compounds. Compounds **9a** and **8a** formed by the addition of amines with the 4-methylphenyl ring and 4-nitrophenyl ring to the 6-methyl-2-thioxo-1,2,3,4-tetrahydropyrimidine-5-yl methanone moiety are more potent than compound **7a** formed by the addition of aniline. The 4-nitrophenyl ring in compound **9a** contributed to enhanced calcium channel-blocking activity compared to the 4-methylphenyl ring in compound **9b** and the phenyl ring in compound **9a**. The presence of the methylphenyl ring in compound **9b** is more effective than the presence of the phenyl ring in compound **9a**, and the existence of a 4-nitrophenyl moiety in compound **8c** is more effective than the presence of the 4-methylphenyl ring in compound **8b**. These results established the importance of the existence of methylphenyl, phenyl, and nitrophenyl rings as pharmacophores for the calcium channel blocker activity.

**FIGURE 2:**
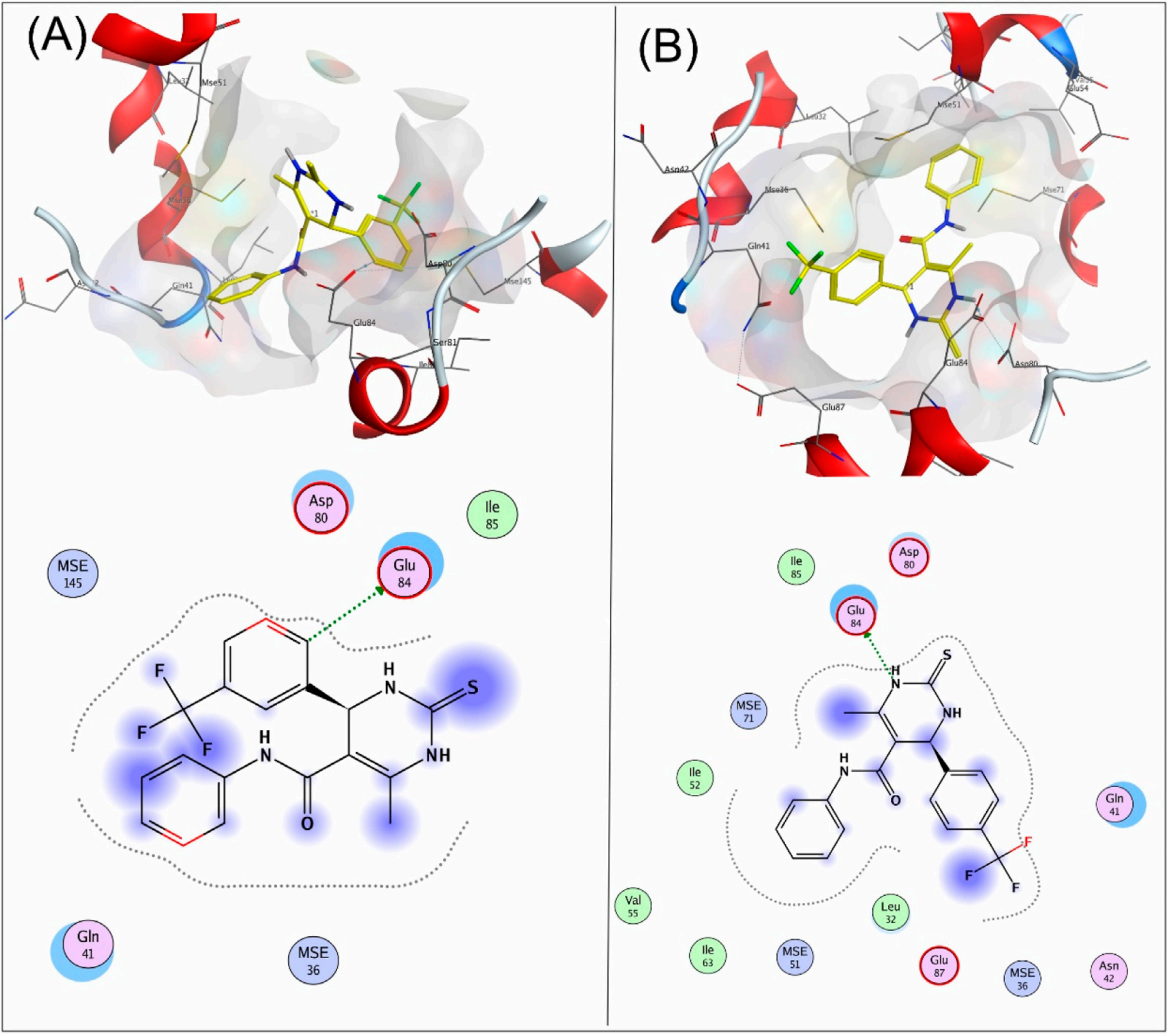
3D and 2D mapping of the **(A)** interaction of compound **8a** and **(B)** compound **9a** with the target protein 6M7H.

Compound **8a** showed a good binding affinity with the target receptor with a docking score of −4.8. The compound shows H-donor interactions with the Glu84 residue of the target protein 6M7H (Figure 2A). Compound **9a** was the most potent inhibitor of all the tested compounds against 6M7H. The compound shows a docking score of −5.7, having H-donor interactions with the Glu84 residue of the receptor (Figure 2B). Compound **9a** shows a comparable docking score and binding affinity with the target protein compared to the standard drug (−5.7 vs. −5.8) (Figure 6).

**FIGURE 3:**
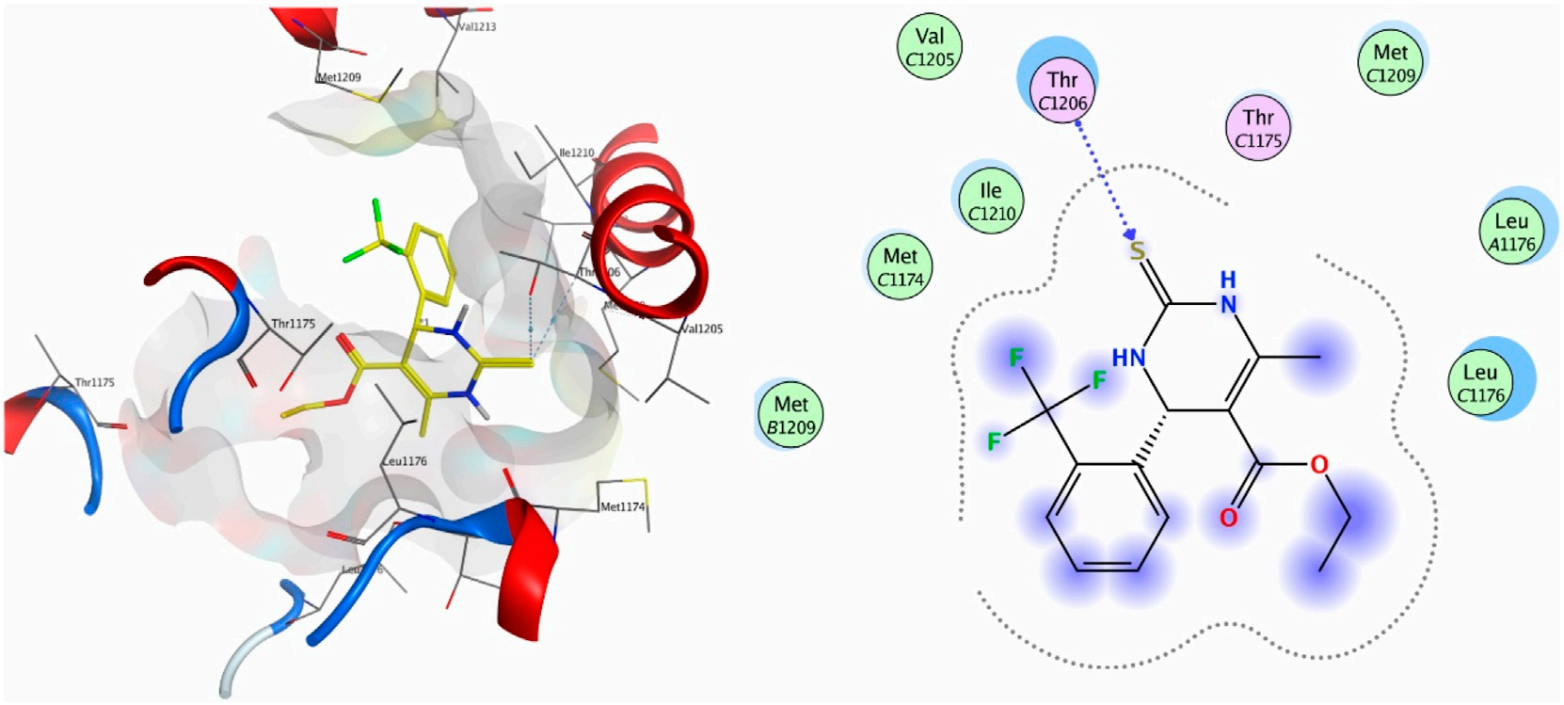
3D interaction map and 2D molecular docking model of compound **4a** with the target protein 4MS2.

When tested against the target protein 4MS2, compound **4a** exhibits H-acceptor interactions with the Thr1206 residue of the target receptor with a docking score of −3.6 and bond distance of 3.35 Å (Figure 3).

**FIGURE 4:**
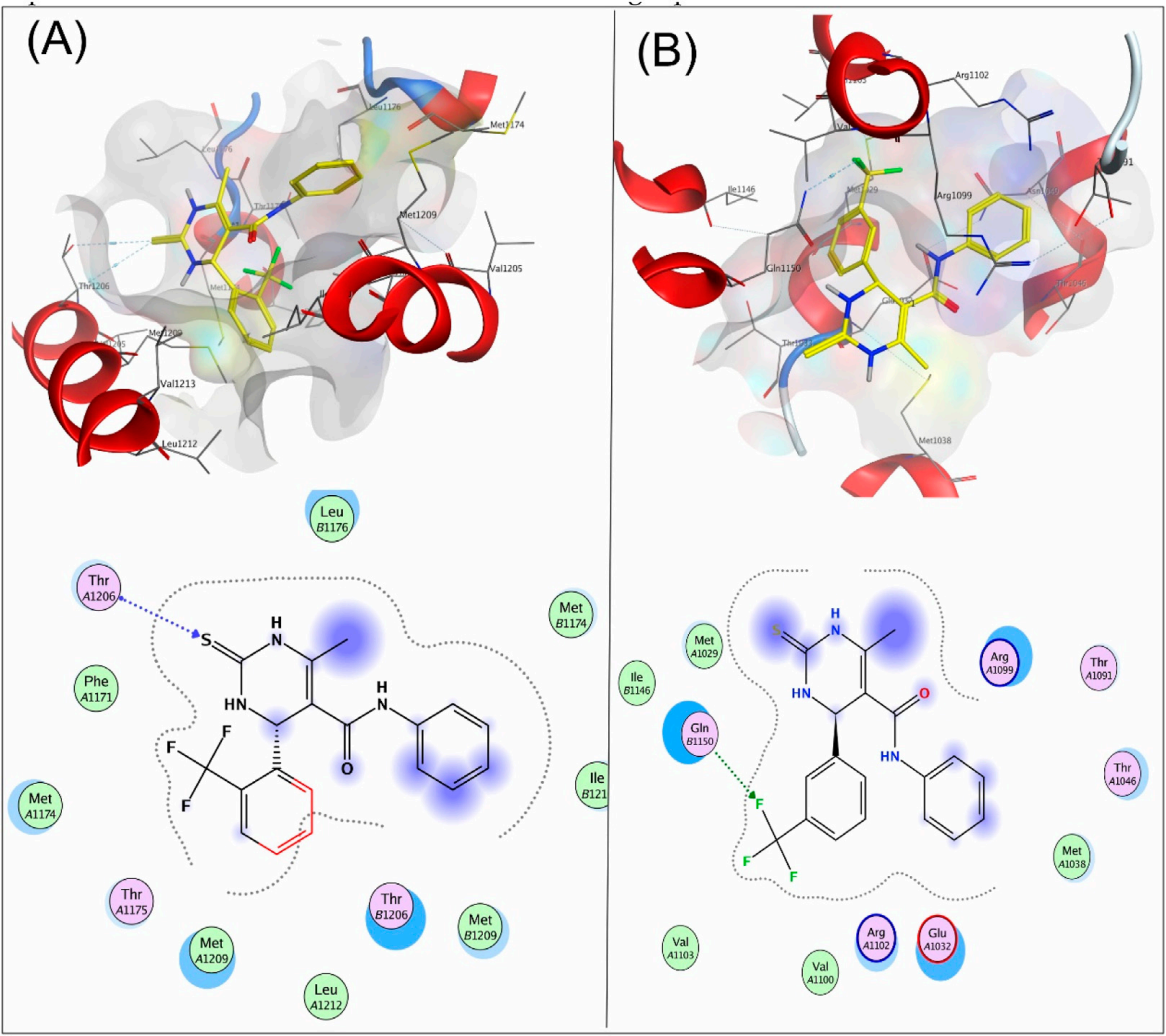
3D interaction map and 2D molecular docking model of compound **7a** with the target protein 4MS2 **(A)** and 3D interaction map and 2D molecular docking model of compound **8a** with the target protein 4MS2 **(B)**.

Figure 4 shows the 3D and 2D interactions of **7a** and **8a** with the target receptor 4MS2. Compound **7a** showed a docking score of −6.2, possessing H-accepter interactions with the Thr1206 residue of the target protein. Compound **8a** showed a docking score of −3.6 and exhibited H-acceptor interactions with the Gln1150 residue of the target protein.

**FIGURE 5:**
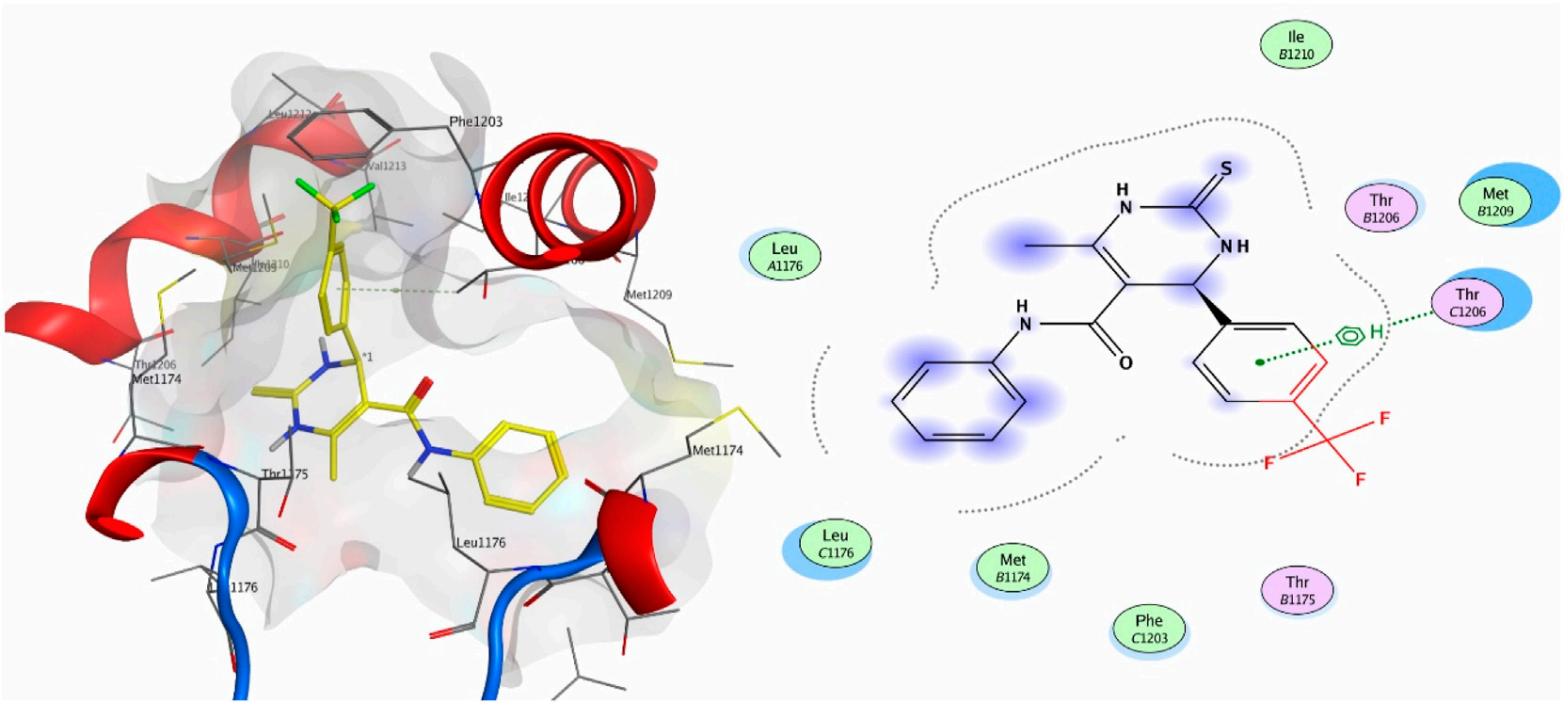
3D interaction map and 2D molecular docking model of compound **9a** with the target protein 4MS2.

**FIGURE 6:**
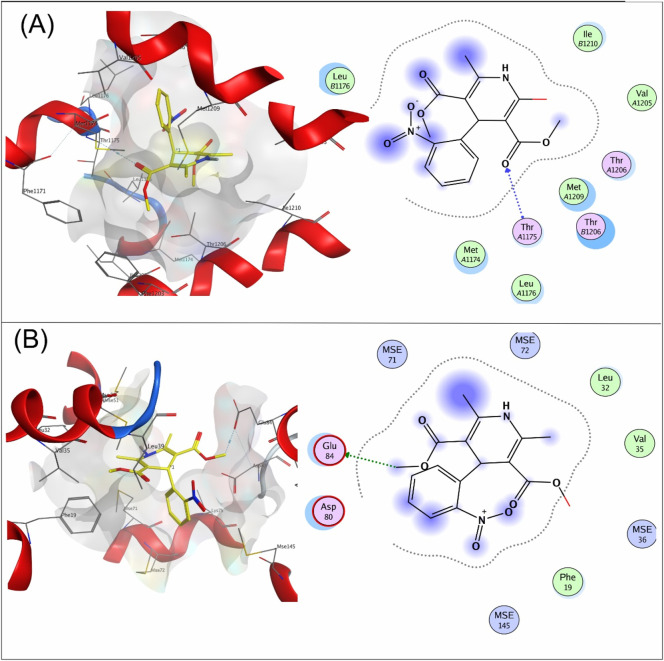
3D interaction map and 2D molecular docking model of the reference drug nifedipine with target proteins 4MS2 **(A)** and 6M7H **(B)**.

Compound **9a** was the most potent inhibitor of all the tested compounds against 4MS2. The compound showed a docking score of −6.3. The compound showed pi–H interactions with the Thr1206 residue of the target protein (Figure 5). Compound **9a** showed a better docking score and binding affinity with the target protein than the standard drug (−5.7), as shown in Figure 6. The target proteins were docked with the reference drug. The reference drug exhibited a docking score of −5.7 and −5.8 with receptor proteins 4MS2 and 6M7H, respectively. As a whole, in 6M7H, residues such as MSE71, Glu84, Asp80, and Glu84 frequently interact with ligands. For 4MS2, residues such as Thr1206, Met1209, and Thr1175 are crucial. Every step in the reaction scheme increased the interacting sites, which increased the docking scores.

## 4 Conclusion

The observed trends in calcium channel-blocking (CCB) activity highlight the critical influence of substitution patterns and molecular modifications on drug efficacy. This study successfully synthesized, characterized, and evaluated nifedipine isosteres with DHP rings for their antihypertensive and CCB activities in rats. The synthesized compounds incorporated –CF_3_ substitutions at the ortho, meta, and para positions of benzaldehyde along with amide-linked derivatives featuring different aromatic substituents. Ortho-substituted derivatives demonstrated superior inhibition than their meta- and para-substituted counterparts, likely due to enhanced molecular interactions with calcium channels. Additionally, amide-linked derivatives **(7a-c** to **9a-c)** exhibited stronger receptor affinity than their ester-linked precursors, suggesting that the –CONH– linkage improves binding efficiency. Among all the tested compounds, compound **9a** emerged as the most potent inhibitor, showing a high docking score (−6.3) and strong H-acceptor and pi–H interactions with key receptor residues (Glu84 of 6M7H and Thr1206 of 4MS2 receptors).

From a biological perspective, these findings underscore the potential of structural modifications in optimizing calcium channel blockers for antihypertensive therapy. By integrating synthetic chemistry, biological evaluation, and computational modeling, this study provides valuable insights into the rational design of next-generation DHP-based CCBs, paving the way for the development of more selective and potent cardiovascular drugs with improved therapeutic profiles.

## Data Availability

The original contributions presented in the study are included in the article/Supplementary Material, further inquiries can be directed to the corresponding author.
