# Supplementary material for: Development of nifedipine isosteres: an integrated approach to the design, synthesis, and biological assessment of calcium channel blockers
Source: Front Chem. 2025 May 12;13:1581037. doi: 10.3389/fchem.2025.1581037 (PMC12104290; doi:10.3389/fchem.2025.1581037)
Supplement: Supplementary file 1 [file DataSheet1.pdf]

^{1}H NMR samples

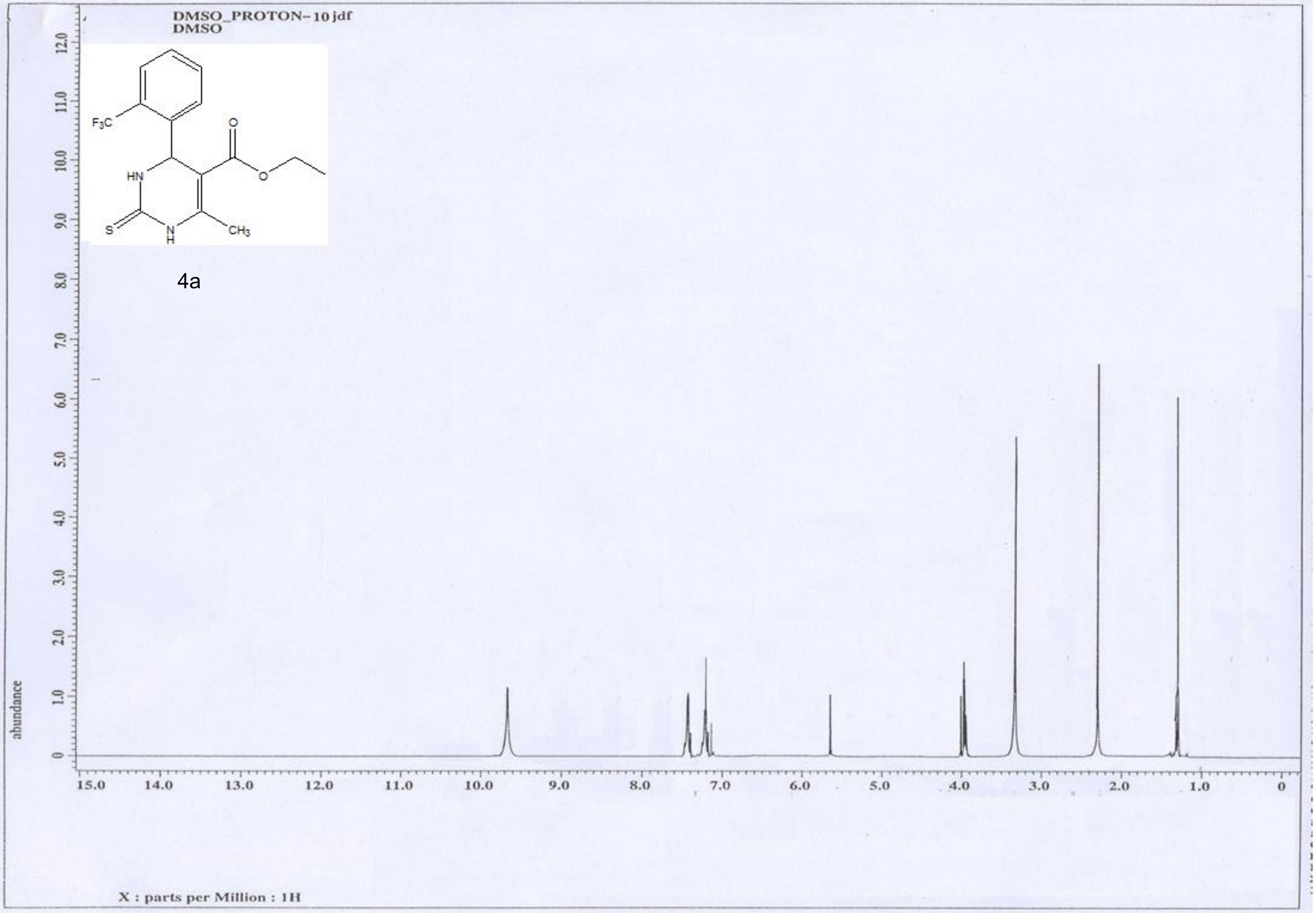
DMSO_PROTON-10 jdf
DMSO
F3C
O
HN
O
S
N
H
CH3
4a
abundance
X : parts per Million : 1H
15.0 14.0 13.0 12.0 11.0 10.0 9.0 8.0 7.0 6.0 5.0 4.0 3.0 2.0 1.0 0
0 1.0 2.0 3.0 4.0 5.0 6.0 7.0 8.0 9.0 10.0 11.0 12.0

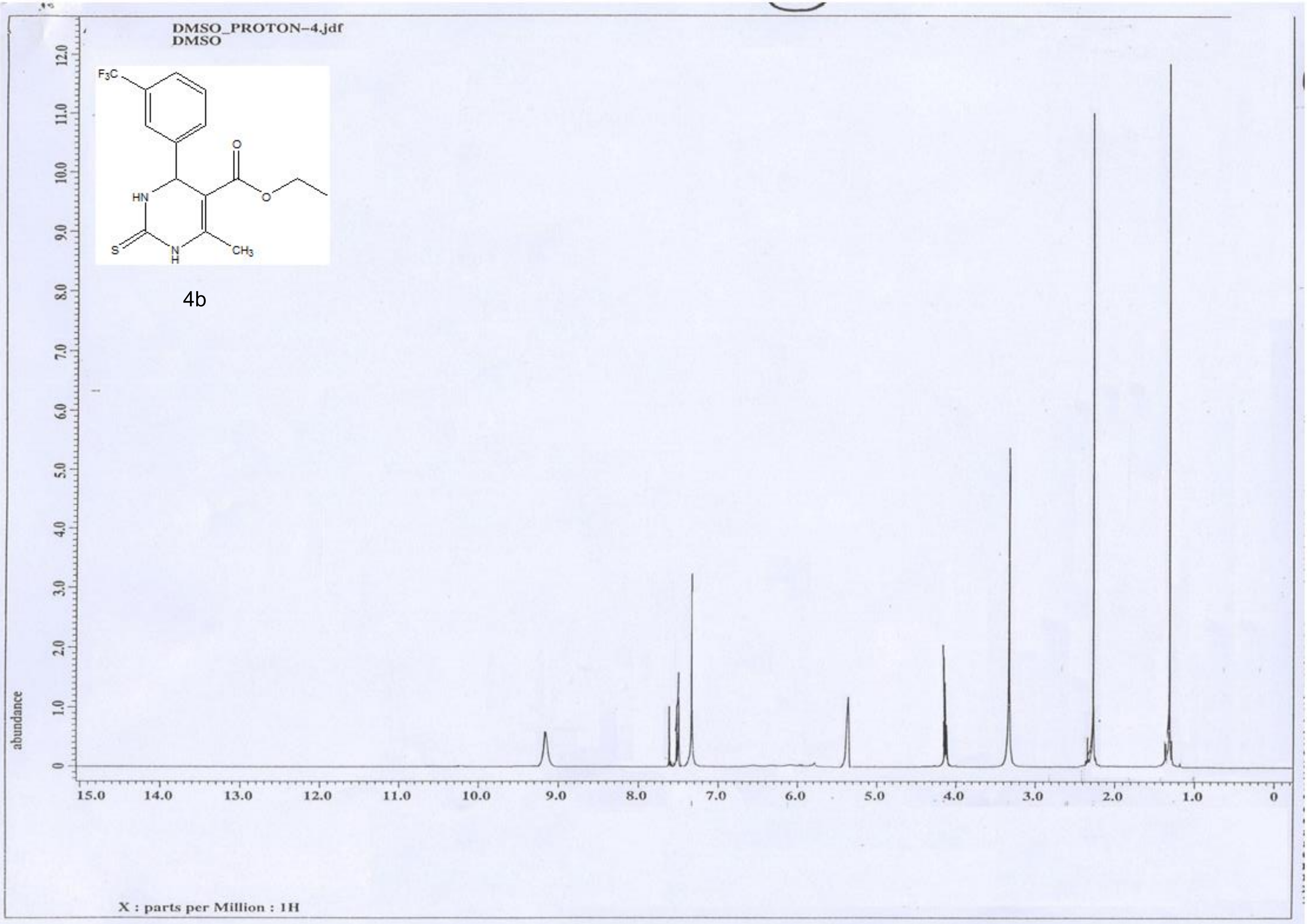
DMSO_PROTON-4.jdf
DMSO
F3C
O
HN
O
S
N
H
CH3
4b
abundance
0
1.0
2.0
3.0
4.0
5.0
6.0
7.0
8.0
9.0
10.0
11.0
12.0
15.0
14.0
13.0
12.0
11.0
10.0
9.0
8.0
7.0
6.0
5.0
4.0
3.0
2.0
1.0
0
X : parts per Million : 1H

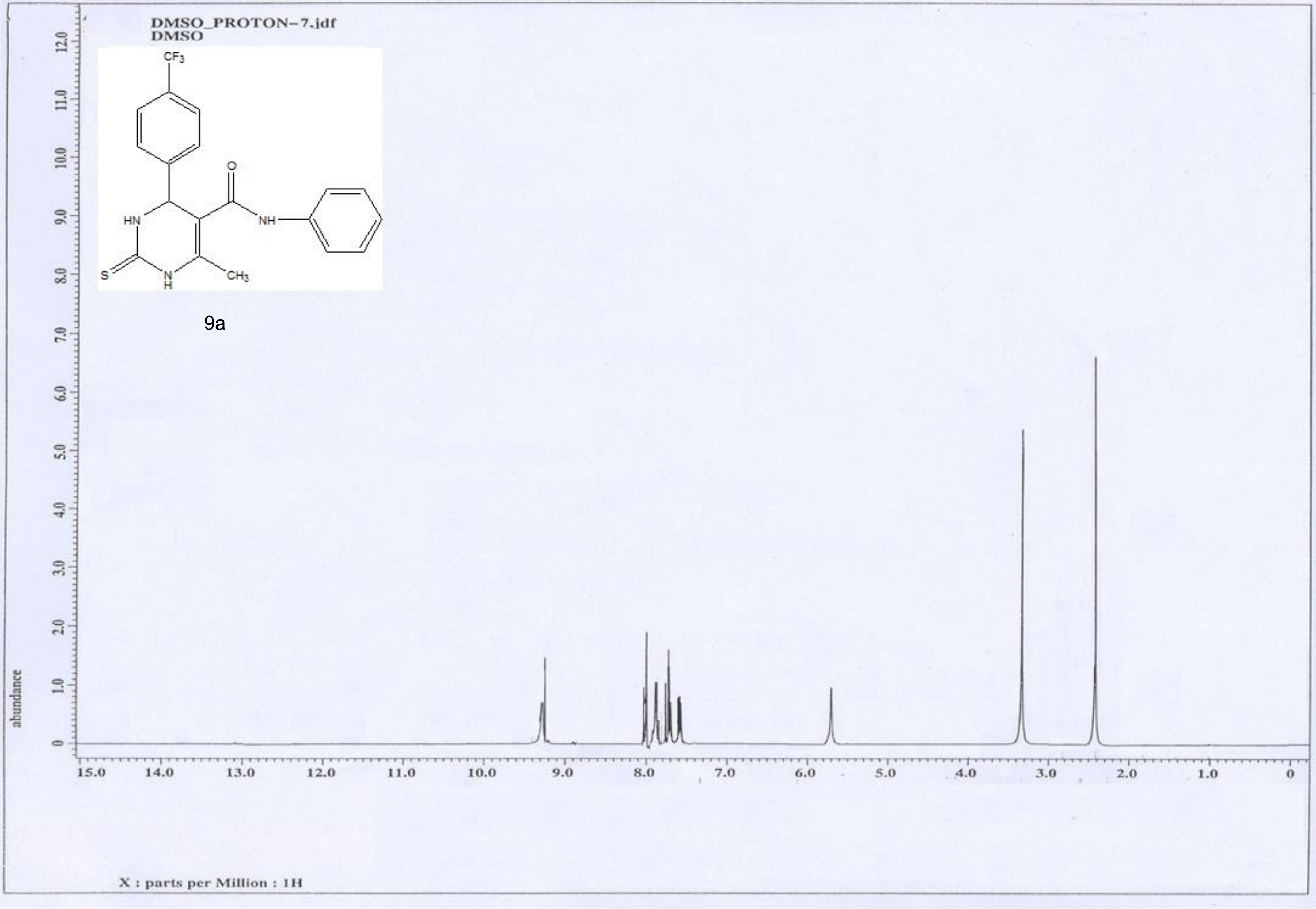
DMSO_PROTON-7.jdf
DMSO
CF3
O
HN
NH
S
N
H
CH3
9a
abundance
X : parts per Million : 1H
15.0 14.0 13.0 12.0 11.0 10.0 9.0 8.0 7.0 6.0 5.0 4.0 3.0 2.0 1.0 0
0 1.0 2.0 3.0 4.0 5.0 6.0 7.0 8.0 9.0 10.0 11.0 12.0

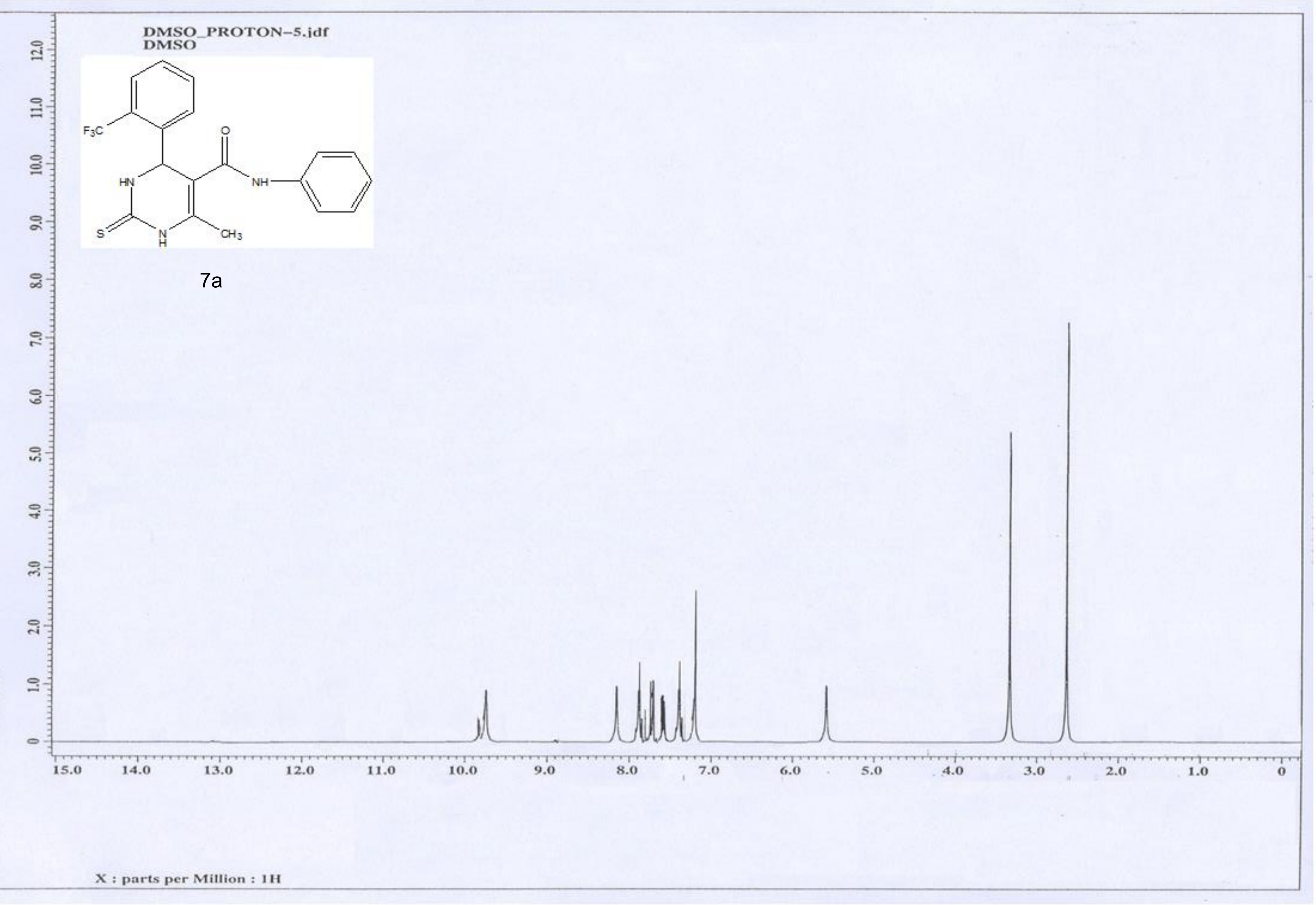
DMSO_PROTON-5.jdf
DMSO
F3C
O
HN
NH
S
N
H
CH3
7a
X : parts per Million : 1H
15.0
14.0
13.0
12.0
11.0
10.0
9.0
8.0
7.0
6.0
5.0
4.0
3.0
2.0
1.0
0

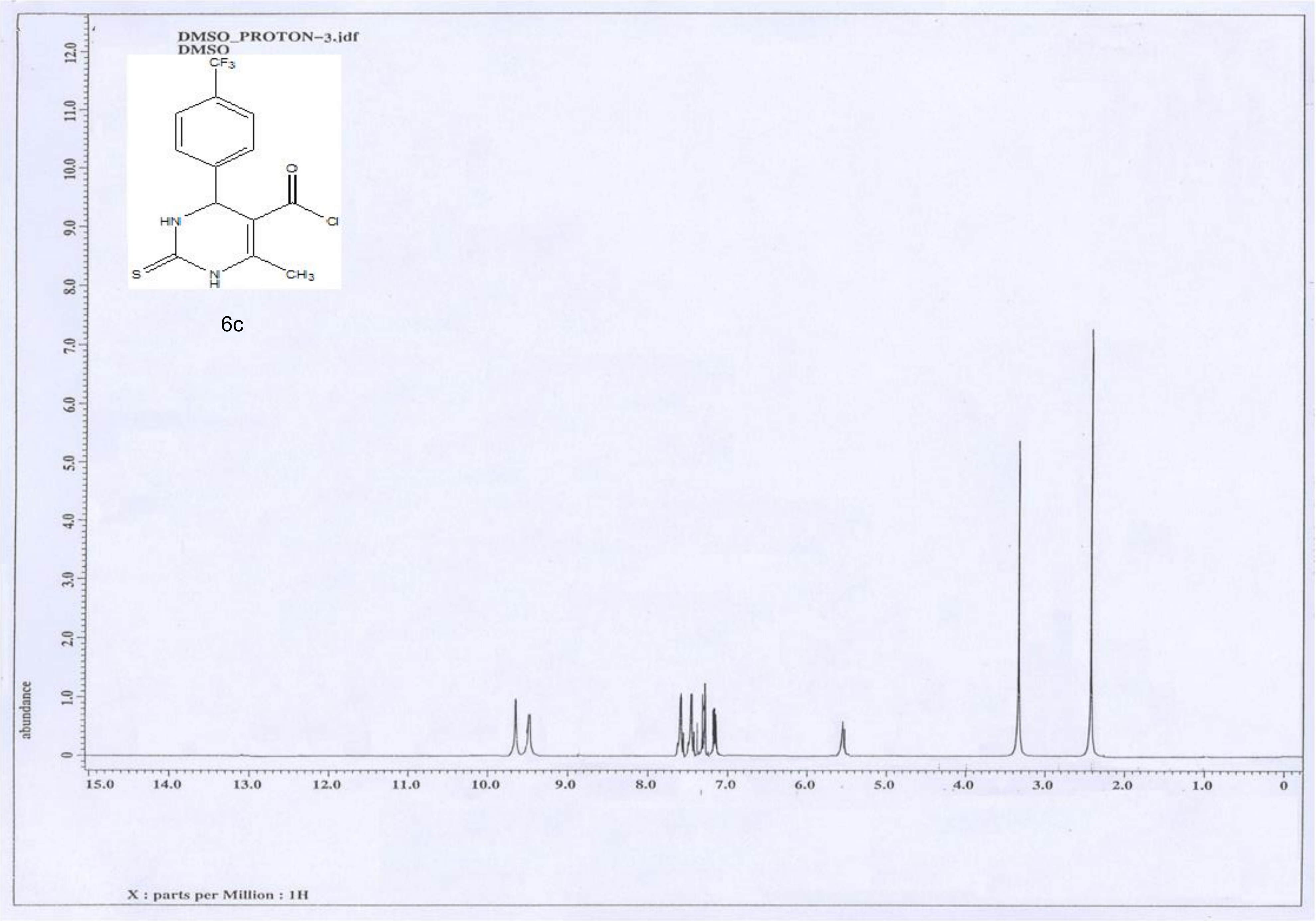
DMSO_PROTON-3.jdf
DMSO
CF3
O
Cl
HN
S
N
H
CH3
6c
abundance
X : parts per Million : 1H
15.0
14.0
13.0
12.0
11.0
10.0
9.0
8.0
7.0
6.0
5.0
4.0
3.0
2.0
1.0
0

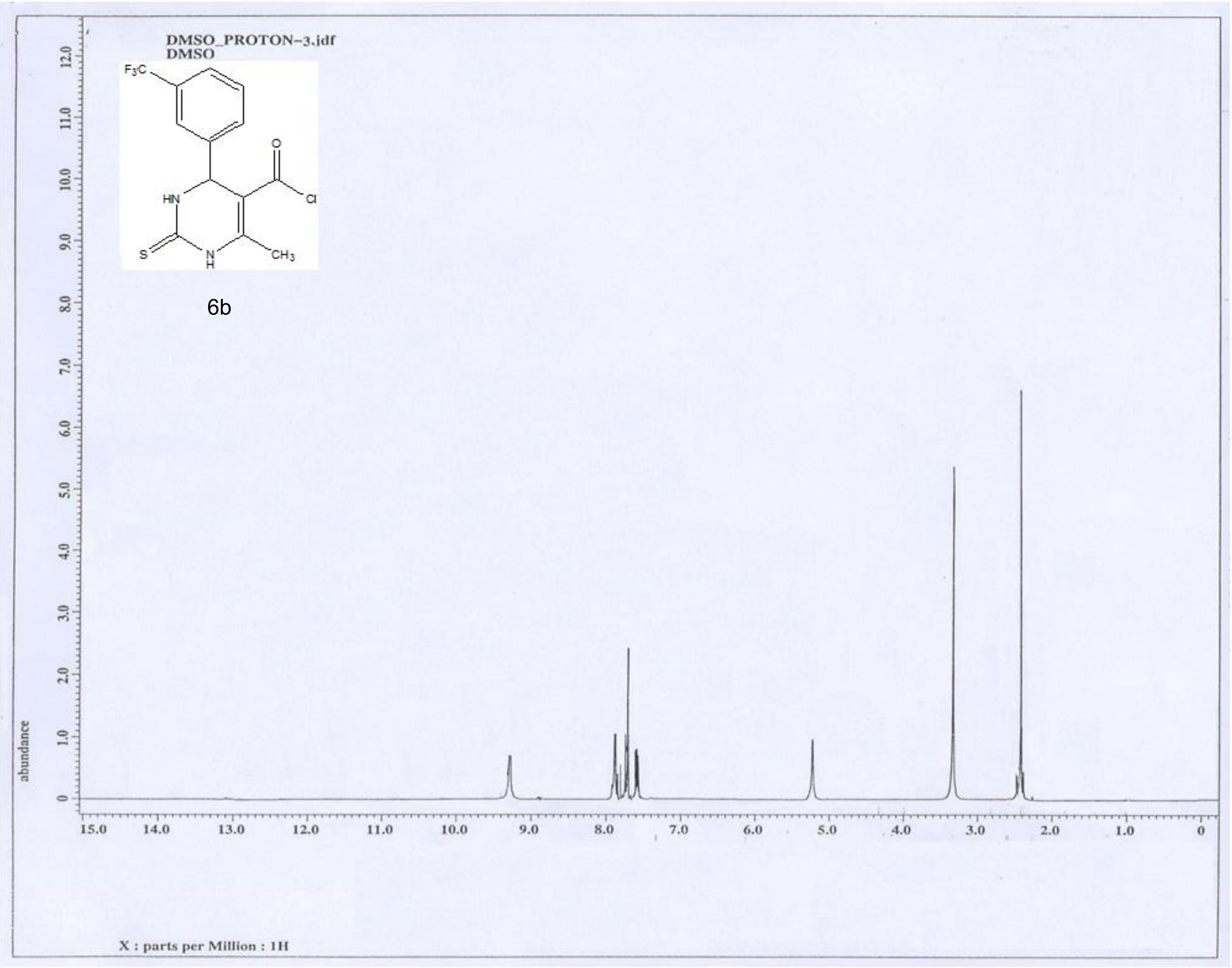

DMSO_PROTON-3.jdf
DMSO
F_3C
HN
O
Cl
S
N
H
CH_3
6b
abundance
X : parts per Million : 1H

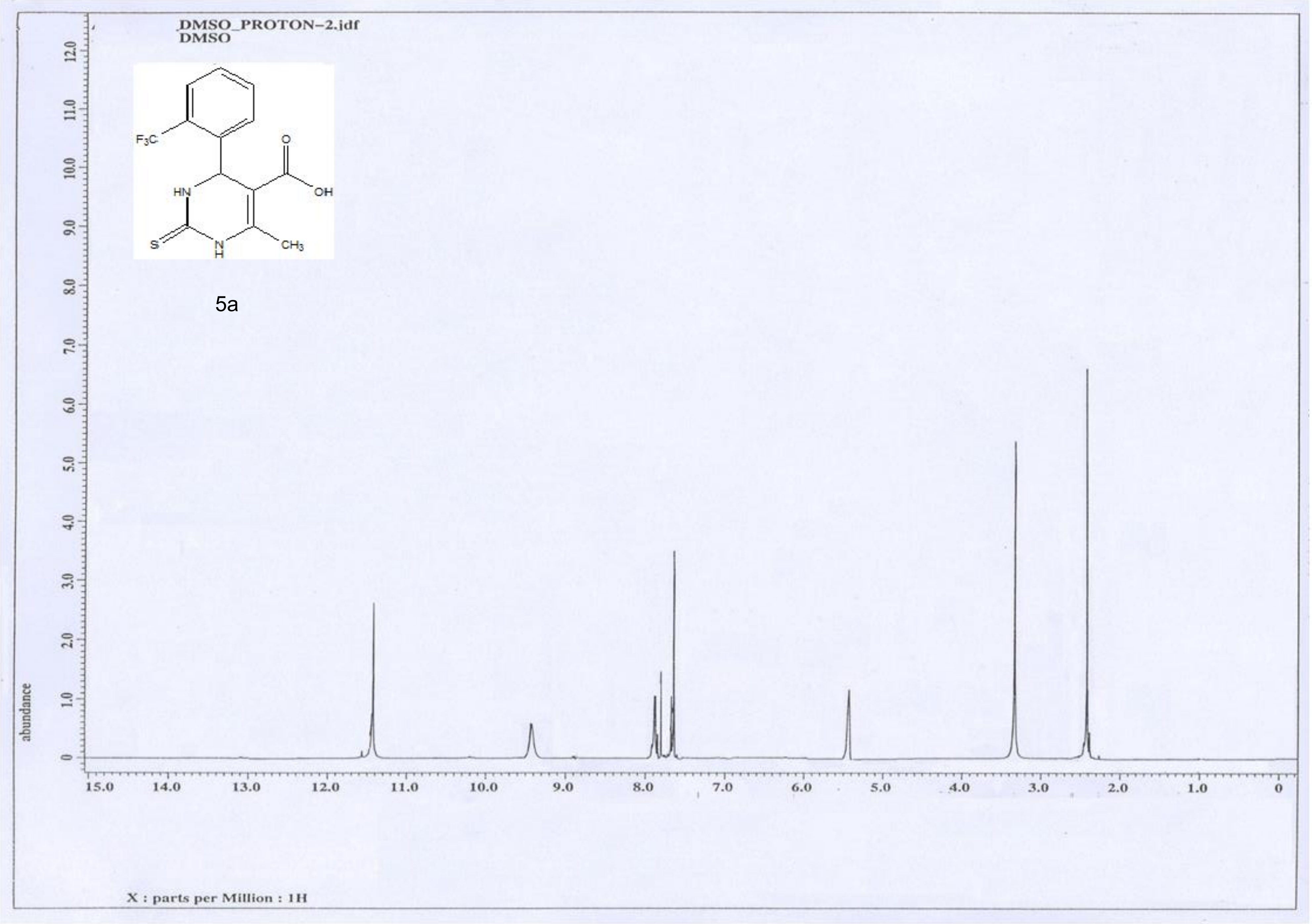
DMSO_PROTON-2.jdf
DMSO
F3C
O
OH
HN
S
N
H
CH3
5a
abundance
X : parts per Million : 1H

^{13}C NMR samples

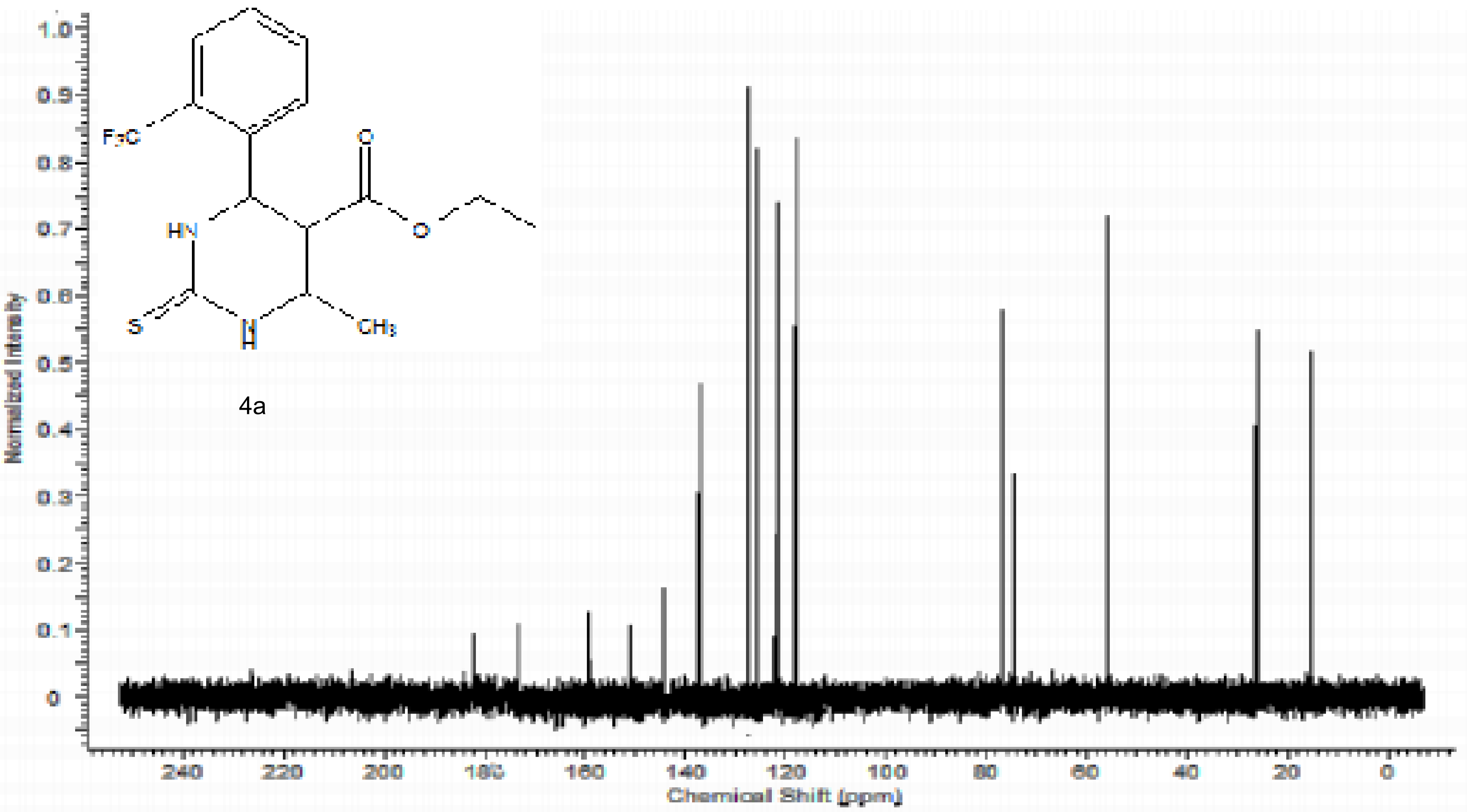

F3C
O
HN
O
S
N
H
CH3
4a
Normalized Intensity
1.0
0.9
0.8
0.7
0.6
0.5
0.4
0.3
0.2
0.1
0
240
220
200
180
160
140
120
100
80
60
40
20
0
Chemical Shift (ppm)

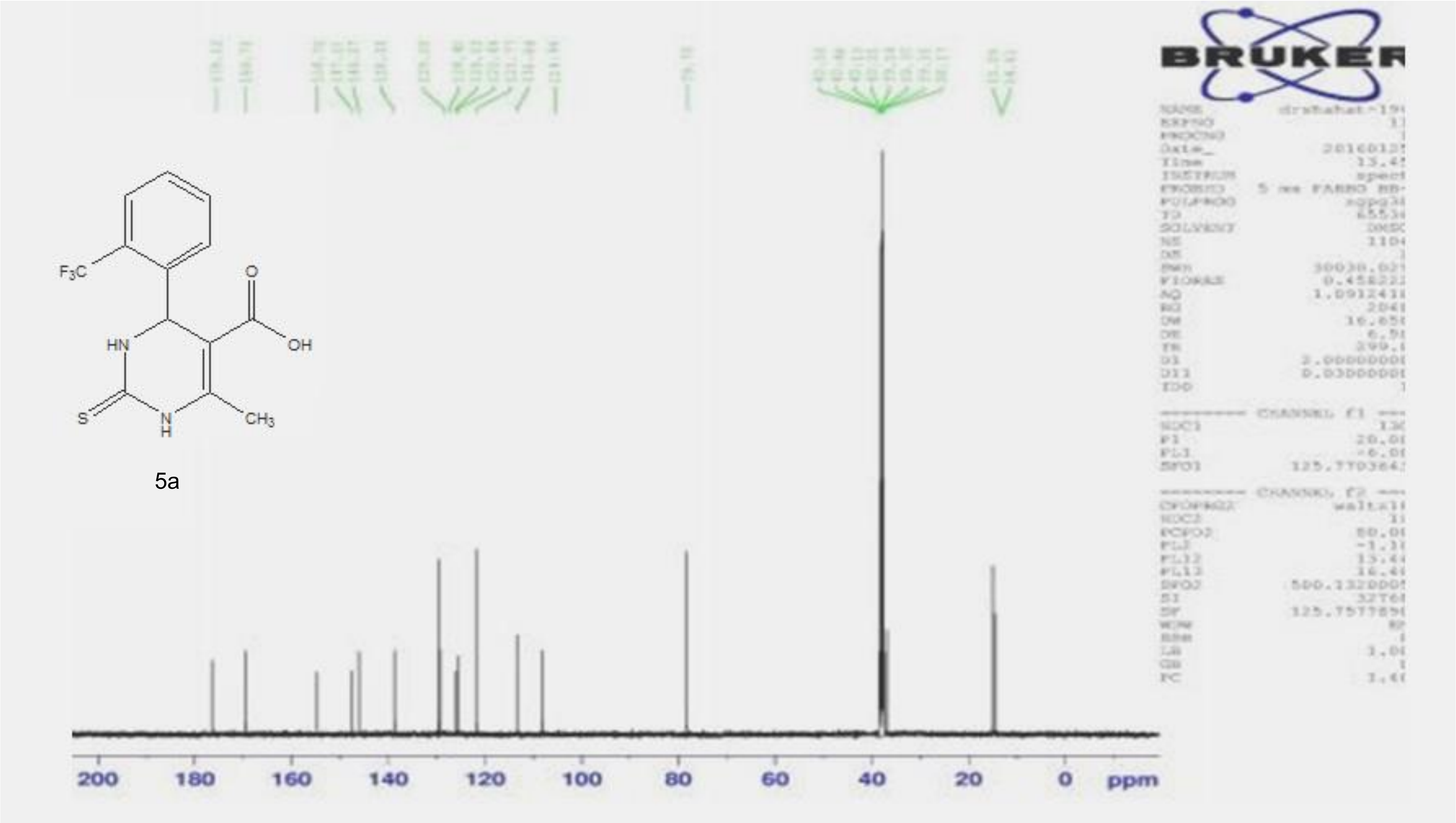
F3C
O
OH
HN
S
N
H
CH3
5a
200
180
160
140
120
100
80
60
40
20
0
ppm

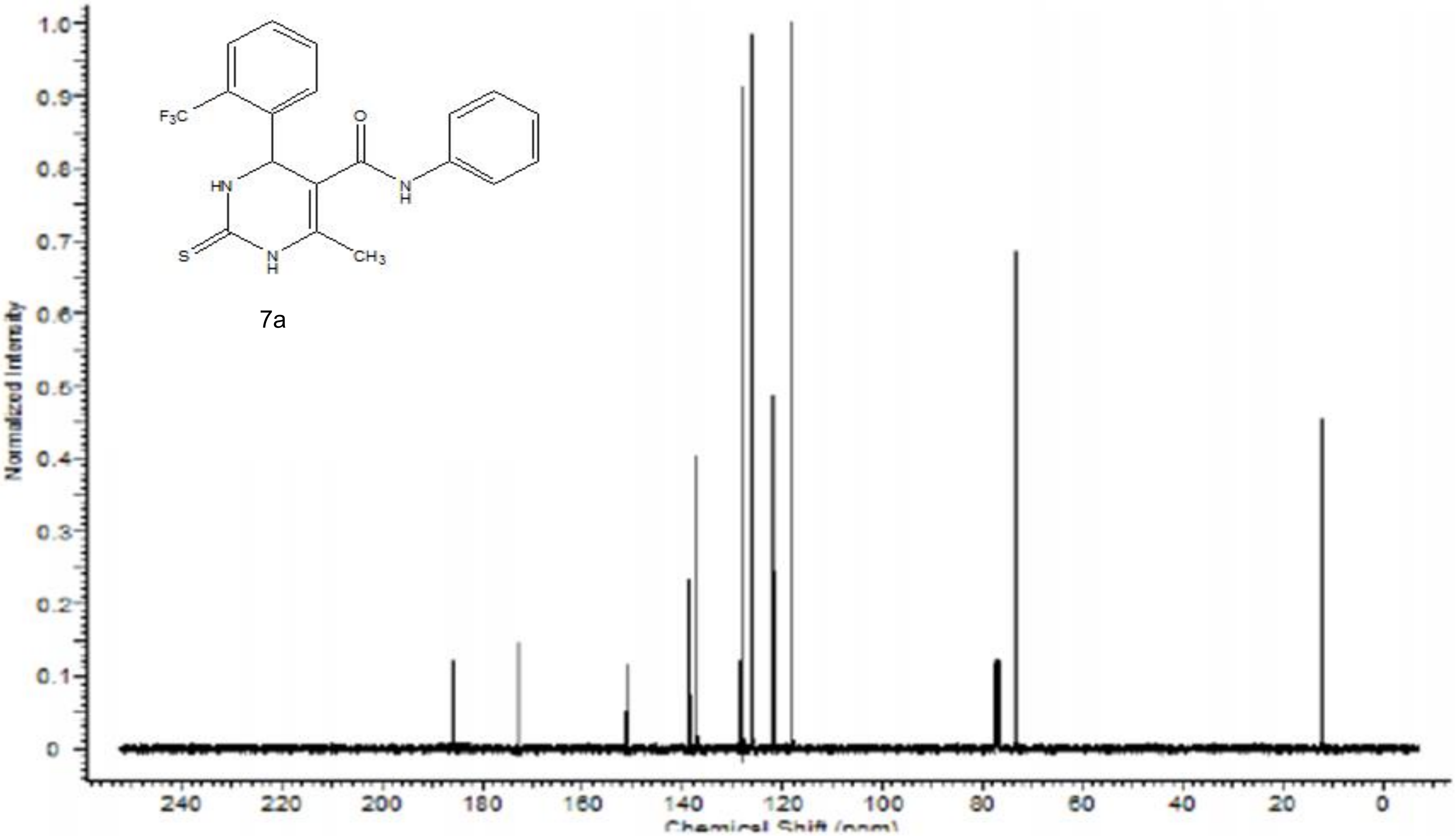
7a
F3C
O
HN
N
H
S
CH3
Normalized Intensity
1.0
0.9
0.8
0.7
0.6
0.5
0.4
0.3
0.2
0.1
0
240
220
200
180
160
140
120
100
80
60
40
20
0
Chemical Shift (ppm)

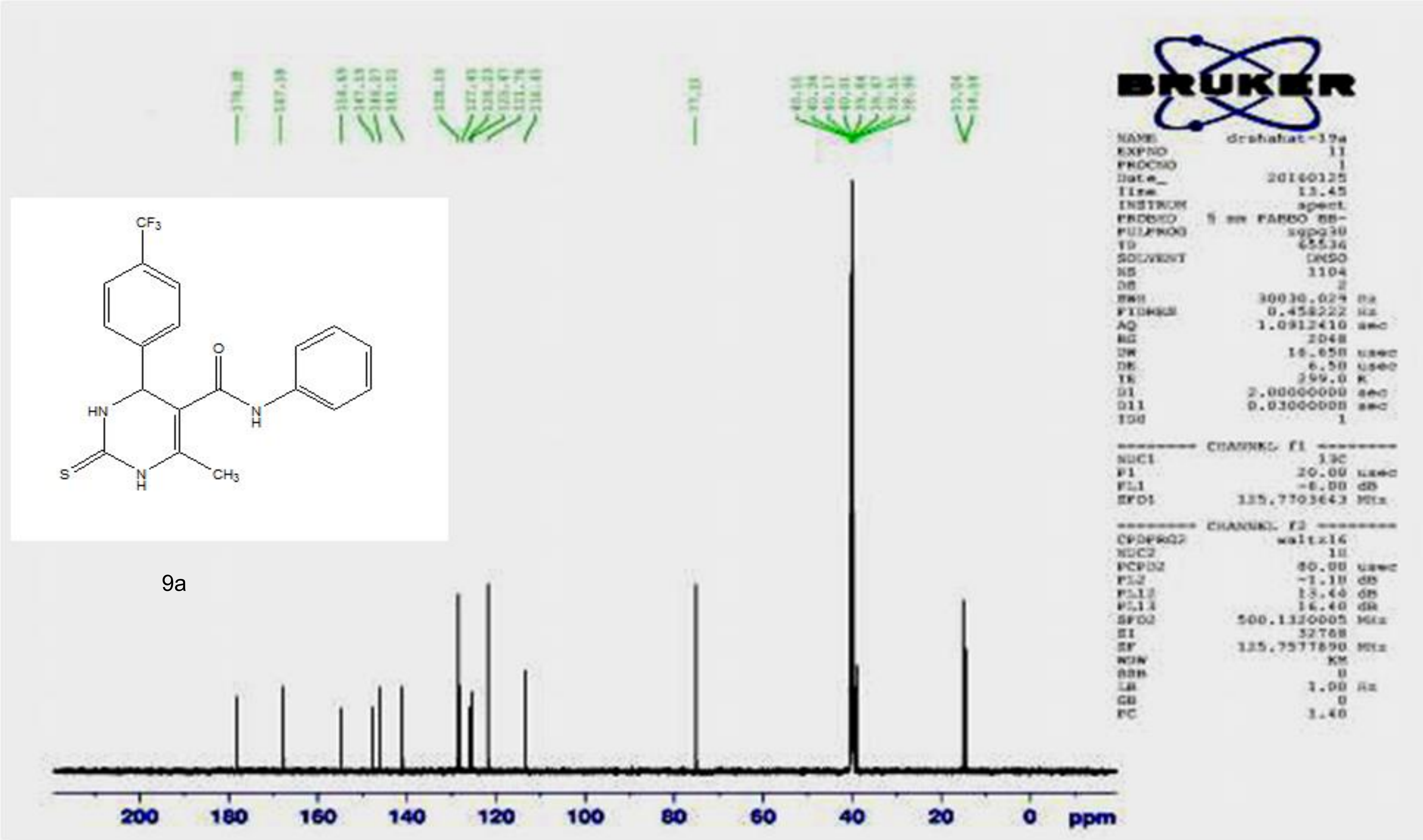
BRUKER
CF3
O
HN
N H
S
N H
CH3
9a
200
180
160
140
120
100
80
60
40
20
0
ppm
